# Neuroligin 3 highlights the sexually dimorphic neural substrates of Drosophila social spacing

**DOI:** 10.1093/genetics/iyag243

**Published:** 2026-09-08

**Authors:** J Wesley Robinson, Abigail T Bechard, Micah R Evans, Judy Kurbaj, Reza Ataei, Khadijat O Mosuro, Sanjali Pillay, Daisy S Lin, Avneet Sahota, Jade N de Belle, Gregory I Robinson, Ryley T Yost, Amanda J Moehring, Anne F Simon

**Affiliations:** Department of Biology, University of Western Ontario, 1151 Richmond Street, London, Ontario N6A 5B7, Canada; Department of Biology, University of Western Ontario, 1151 Richmond Street, London, Ontario N6A 5B7, Canada; Department of Biology, University of Western Ontario, 1151 Richmond Street, London, Ontario N6A 5B7, Canada; Department of Biology, University of Western Ontario, 1151 Richmond Street, London, Ontario N6A 5B7, Canada; Department of Biology, University of Western Ontario, 1151 Richmond Street, London, Ontario N6A 5B7, Canada; Department of Biology, University of Western Ontario, 1151 Richmond Street, London, Ontario N6A 5B7, Canada; Department of Biology, University of Western Ontario, 1151 Richmond Street, London, Ontario N6A 5B7, Canada; Department of Biology, University of Western Ontario, 1151 Richmond Street, London, Ontario N6A 5B7, Canada; Department of Biology, University of Western Ontario, 1151 Richmond Street, London, Ontario N6A 5B7, Canada; Department of Biology, University of Western Ontario, 1151 Richmond Street, London, Ontario N6A 5B7, Canada; Department of Biological Sciences, University of Lethbridge, 4401 University Drive, Lethbridge, Alberta T1K 3M4, Canada; Department of Biology, University of Western Ontario, 1151 Richmond Street, London, Ontario N6A 5B7, Canada; London Health Sciences Centre Research Institute, 800 Commissioners Rd E, London, Ontario N6A 5W9, Canada; Department of Biology, University of Western Ontario, 1151 Richmond Street, London, Ontario N6A 5B7, Canada; Department of Biology, University of Western Ontario, 1151 Richmond Street, London, Ontario N6A 5B7, Canada

**Keywords:** Drosophila, social space, social behavior, neural substrates, *neuroligin*, mushroom bodies, *fruitless*, acetylcholine, GABA, dopamine, and dopamine receptors

## Abstract

In *Drosophila melanogaster*, Neuroligin 3 (Nlg3) is a postsynaptic membrane protein important for synapse development and social spacing behavior, and its human orthologs are associated with autism. We localized Nlg3 to 3 brain structures: the mushroom bodies (MB) calyx, the optic lobes (OL), and the protocerebral bridge (PB). RNA knockdown of *nlg3* in each structure independently replicated the effect of knocking down in all *nlg3*-expressing neurons. In contrast, hyperactivating and silencing all *nlg3*-expressing neurons, including those within the MB, PB, and OL, affected social spacing by sex: females were further apart after either manipulation, whereas males’ social space was increased, only after hyperactivation. Decreasing acetylcholine release in the MB via choline acetyltransferase (ChAT)-RNAi caused decreased social space in both sexes. In contrast, RNAi targeting dopamine receptors in the MB increased social space mainly in males. Finally, we also tested *fruitless-*expressing (*fruP1*) neurons, which regulate sexually dimorphic behaviors. Hyperactivating *fruP1* neurons decreased social space in both sexes, while silencing increased social space only in males. These results highlight sex-specific neural substrates for social space and implicate the OL, MB, *nlg3*, and *fruP1* neurons, in this behavior.

## Introduction

Social behaviors are fundamental for communication between individuals, and are influenced by environmental cues that are processed and integrated in the brain. Differences in social communication and interactions are clinical features of autism spectrum disorders (ASD). *Drosophila melanogaster* offers a simple system for studying the neural and genetic basis of social interaction and neurodevelopmental disorders such as ASD (Sokolowski 2001).

Although simpler than those of vertebrates, flies social behaviors reflect the basic principles of social interaction (Sokolowski 2010). Studying how Drosophila integrates social and environmental cues can reveal conserved mechanisms, potentially relevant to ASD (Ueoka et al. 2017; Bellosta and Soldano 2019).

An important component of Drosophila social interactions within a group context is social spacing—the distance between individuals in a group. To assess fly group behavior, we use a social space assay to measure the average number of flies within a 4-body lengths (4BL) radius of each fly (Yost et al. 2020) in the social space chamber (Simon et al. 2012). The measure of number of flies within 4 BL is a metric of social space that assesses clustering. It is the only measurement that takes in account the different body sizes of males and females, as opposed to social space index (Simon et al. 2012), the distance to the closest individual (which assesses only individual preferred distances), or the distribution of distance to all flies (which assesses the overall group density (Castells-Nobau et al. 2019; Jiang et al. 2020). The number of flies within 4BL is also the most conservative: when number of flies within 4BL changes, all other measures of social space also change, while the reverse was not necessarily true (Jiménez-Padilla et al. 2024).

We and other research groups have revealed some of the mechanisms underlying social spacing decisions with this assay (Burg et al. 2013; Fernandez et al. 2017; Xie et al. 2018; Yost et al. 2020). So far, we know that mutations in genes affecting eye function make flies less social, ie increased social space (Simon et al. 2012; Burg et al. 2013; Jiang et al. 2020). Similarly, mutant flies with disrupted gustation and mechanosensation abilities display increased social spacing in both sexes (Jiang et al. 2020). However, in *Orco* mutants, which have reduced olfactory perception, only females’ social space is affected, causing them to settle further apart. This suggests a sensory modality-specific genetic influence on social behavior that can have sex-specific effects (Simon et al. 2012; Jiang et al. 2020).

Other genes expressed in the brain influence Drosophila social space behavior, such as those important for synaptic transmission and synaptogenesis (Burg et al. 2013; Wise et al. 2015; Fernandez et al. 2017; Brenman-Suttner et al. 2019; Hope et al. 2019; Ueoka et al. 2019; Jiang et al. 2020; Kanellopoulos et al. 2020; Yost et al. 2020; Shilpa et al. 2021; Cao et al. 2022). At the synapse level, the concentration of neurotransmitters, which is tightly regulated by biosynthesis enzymes or vesicular packaging proteins, has been shown to affect social spacing. Tyrosine hydroxylase (TH), the rate-limiting enzyme in dopamine synthesis, and the vesicular monoamine transporter, which packages dopamine into synaptic vesicles, both regulate dopamine levels (Deshpande et al. 2020). Modulating the abundance or activity of these proteins influences social spacing in Drosophila, indicating that dopamine plays a role in regulating social space (Fernandez et al. 2017).

Many human orthologs of Drosophila neural genes have also emerged as potential candidates in neuropsychiatric disorders, helping bridge the gap between basic biological processes and complex human pathologies (Wangler et al. 2017; Bellen et al. 2019; Lee 2022). The human gene neurobeachin *(NBEA*) encodes a structural scaffolding postsynaptic density protein and is implicated in ASD (Castermans et al. 2003). In Drosophila, mutations in the *NBEA* homolog *rugose* result in altered fly social spacing, indicating a conservation of function across species (Wise et al. 2015). Furthermore, synaptic proteins Neuroligin and Neurexin, neurotransmission facilitators that aid in synapse formation and receptor recruitment, have been shown to influence social behavior in multiple species, including *D. melanogaster* (Ey et al. 2012; Hahn et al. 2013; Corthals et al. 2017, Yost et al. 2020; Yagishita-Kyo et al. 2021; Qin et al. 2024; review Taylor et al. 2020). In humans, mutations in Neuroligin genes are associated with ASD (Jamain et al. 2003; Südhof 2008). Similarly, in Drosophila, mutations in orthologs of these genes lead to altered social behaviors (Corthals et al. 2017; Yost et al. 2020, Yost et al. 2024).

In Drosophila, Neuroligins (Nlg) are a family of postsynaptic membrane proteins with conserved functions in synapse formation and maintenance. Interactions between Nlgs and presynaptic Neurexins mediate these functions, facilitating cell–cell adhesion and neurotransmission (Südhof 2008). During Drosophila neural development, larval *nlg3* mutants have a higher number of synaptic connections, less glutamate receptors recruitment, and weaker synaptic transmission compared to control flies (Xing et al. 2014). Furthermore, *nlg3* knockout flies display a sexually dimorphic and age-associated effect on fly aggression, social space, and response to isolation (Yost et al. 2020, Yost et al. 2024). Interestingly, *nlg3* deletion decreases only male, but not female aggression; while increasing male social space in 3- to 4-d-old flies, and in both sexes by 7 to 10 d old. However, mutant *nlg3* flies of both sexes had reduced climbing performance (Yost et al. 2020). The sexually dimorphic effects of *nlg3*n and the male-specific differences in dopamine (DA) levels after isolation (Yost et al. 2020; Yost et al. 2024) Yost et al. 2024) indicate that within similar social environments, males and females make the same decisions (increased social spacing) but using different neural substrates. These data, obtained through manipulating *nlg3* expression and the social environment, served as an entry point for the present work: we further investigate the cells expressing Nlg3, identified for the first time in this study, and start to decipher the sexually dimorphic role of those Nlg3 cells.

Sex-specific behaviors in Drosophila, such as male courtship, female receptivity, and some social interactions are regulated by *fruitless* (*fru*) neurons (Lee and Hall 2000; Vrontou et al. 2006; Wu et al. 2019; Chowdhury et al. 2020; Brovkina et al. 2021). Regulated by multiple promoters each producing unique transcripts, the *fruitless* gene encodes a transcription factor important for sex determination in Drosophila, and produces some male-specific transcripts required for the development and function of male-specific neural circuits (Sato et al. 2019). The *fruP1* transcripts undergo sex-specific splicing and contribute to the sexual differentiation of neural circuits that influence behaviors such as courtship and aggression (Sato et al. 2019). Additionally, transcripts *p2-p5* are necessary for female receptivity, adult viability and fly morphology, with the last 2 lacking sex-specific effects (Sato and Yamamoto 2019; Chowdhury et al. 2020). Widespread interneurons express *fru* in the adult fly brain and ventral nerve cord; some *fru* neurons release acetylcholine, GABA, and glutamate, while other *fru* neurons also release dopamine, serotonin, tyramine, and octopamine (Palmateer et al. 2023). Notably, male social spacing is altered when *fru* expression is knocked down in the oenocytes (Sun et al. 2023).

Finally, the mushroom bodies (MB), also known to play a role in Drosophila social spacing (Burg et al. 2013), are well-studied invertebrate brain structures primarily involved in fly olfactory learning and memory. The MB are comprised of approximately 2,200 neurons (Aso et al. 2014; Li et al. 2020), and their unusual, yet highly structured shape suggests a specialized organization and function (Davis 2023; Lin 2023). The MB display structural flexibility in response to social isolation and are the central processing structure of the Drosophila brain (Davis 2023). Consisting of densely packed neurons called Kenyon cells, the MB integrate many sources of sensory information to modulate fly behavioral responses (Parnas et al. 2024; Suárez-Grimalt et al. 2024). Mutating *narrow abdomen* (*na*), an ion channel with strong expression in the MB, increased social space; an effect that was rescued by expressing non-mutant *na* in cholinergic neurons, especially those of the MB (Burg et al. 2013).

While progress has been made in identifying individual genes, synaptic proteins, and brain structures that influence Drosophila social spacing, these factors have yet to be integrated to form a working model of social spacing neural circuitry. Based on Nlg3 localization and the sex-specific roles of certain brain regions in social spacing, we elucidated here some of the neural substrates that directly influences social spacing behavior.

## Materials and methods

### Fly stocks and husbandry

All flies were reared in mixed-sex bottles containing our standard food (brown sugar, corn meal, yeast, agar, benzoic acid, methyl paraben, and propionic acid, in proportions like those of Jazz-Mix from FisherBrand). During development and aging, flies were kept at 50% relative humidity, 25 °C, and on a 12:12 h light:dark cycle. Parents were removed after 7 d of egg laying resulting in a maximum parental age of 14 d, because parental age affects offspring social spacing (Brenman-Suttner et al. 2018). The Canton-S (Cs) control line came from Seymour Benzer in 1998. *Gal4* driver lines were outcrossed 5 times into Cs to minimize behavioral variation due to genetic background. Apart from the *UAS-nlg3* RNAi that we created (see below), UAS-linked RNAi effector lines came from the Bloomington Drosophila Stock Center through the Transgenic RNAi Project (TRiP) at Harvard Medical School. These RNAi lines share a common genetic background, hereafter referred to as the *attP* background after the corresponding control line (attP2). All genotypes used are reported in Supplementary Table 1.

Crossing schemes used to create the appropriate experimental treatment and genetic controls in either the *attP* lines or the Cs backgrounds can be found in the Supplementary material (Supplementary Fig. 1a and b). The results of the statistical analysis obtained including all the UAS effector lines and drivers, despite being in a different genetic background, are presented in Supplementary Table 4 and Supplementary Figs. 9–12, where the effect of genetic background may be observed in some experiments. It is typical to compare the Gal4>UAS lines to both the Gal4/+ and the UAS/+ lines to ensure that the Gal4 driver is not toxic, and that the UAS-line is not leaky, but the conclusions of those comparisons are misleading when the lines are not all in the same genetic background. In our experiments, as we used many lines from diverse genetic background, we outcrossed a subset of them and only compared controls that shared the same genetic background. But our results still allow us to demonstrate that the Gal4 lines that were tested as heterozygotes are not toxic: *nlg3*-Gal4 in Fig. 1; MB in Supplementary Figs. 11–13; PB and OL in Supplementary Fig. 12, *fru P1*-Gal4 in Supplementary Fig. 14. Furthermore, Fig. 1 shows that there is no leaky effect of the UAS *nlg3*-RNAi. The other UAS lines were used without *dicer*, and as heterozygotes, which limits the leakiness of the UAS-RNAi. By comparing the UAS/+ with the Gal4>UAS line we can distinguish between effect of the UAS alone and the UAS driven by Gal4; this is what is shown in the main text for most experiments, apart from the MB>DopR figure. But, in that case, Supplementary Fig. 13 shows no difference in conclusions when also compared to the UAS/+, even though it is in a different genetic background.

### Confirming the *Gal4* driver lines

The MB, PB, and OL *Gal4* driver lines we used were part of a collection generated for neurobiology research at Janelia farm (Jenett et al. 2012). Their expression pattern can be seen on the FlyLight website (https://www.janelia.org/project-team/flylight and Meissner *et al.* 2023), and the line number for the FlyLight database is indicated in Supplementary Table 1. The *FruP1-Gal4* driver was created by Stockinger et al. (2005, and Supplementary Table 1). Prior to performing assays, we ensured that the lines we received were indeed driving expression as reported. We confirmed the reported expression by dissecting and assessing the fluorescence of whole adult brains driving membrane bound GFP (Supplementary Data and Supplementary Fig. 2).

### Generating the UAS-*nlg3* RNAi line

The shRNA sequences targeting *nlg3* mRNA were obtained by using the model provided by Vert et al. (2006). The RNAi targeted sequence is complementary to the first coding exon of the *nlg3* transcript. The length of the *nlg3* shRNA is 60 bases for the full hairpin; and the length of the entire pVALIUM20 plasmid containing the shRNA is ∼7.8 kbp. The hairpin sequence used is: 5′ CAGCAACTCGAACTCGAACTAtagttatattcaagcataTAGTTCGAGTTCGAGTTGCTG 3′

Within the hairpin sequence, uppercase letters represent the nucleotides complementary to the *nlg3* mRNA, while lowercase letters represent the nucleotides that connect the 2 halves of the pin. The shRNA construct was cloned into the vector pVALIUM20 (vermillion eye color maker, (Ni et al. 2011) using the protocol in Chang et al. (2014). DNA was injected into genotype *y v, nanos-integrase, attP40/attP40* (*y[1] v[1] P{y[+t7.7]=nos-phiC31\int.NLS}X; P{y[+t7.7]=CaryP}attP40*—Bloomington Stock #: 25709). Progeny were mated to the balanced line *w[1118]/Dp(1;Y)y[+]; CyO/nub[1] b[1] sna[Sco] lt[1] stw[3]; KRS/TM6B, Tb[1]* (Bloomington Stock #: 3707). Details of this protocol can be found in Isaacson (2018).

Then, offspring containing the transgene were outcrossed 5 times into the Cs control background.

### Confirming efficacy of the UAS-*nlg3* RNAi construct using RT-qPCR

Heads from ∼20 male or female flies (3 to 5 d old) were collected separately. Control and RNAi treatment groups used in the behavioral assays were genotype and age-matched, ensuring consistency across experiments. Total RNA was extracted using TRIzol reagent (#15596026- Invitrogen) according to the manufacturer's instructions. RNA samples were treated with DNase (TURBO DNase-Invitrogen) to eliminate genomic DNA contamination, and cDNA was synthesized using the cDNA Synthesis Kit (#1708841-BIO-RAD). RT-qPCR was performed using the PowerUp SYBR Green Master Mix (Thermo Fisher Scientific) on a CFX Connect Real-Time PCR Detection System (BIO-RAD). Relative expression levels were calculated using the ΔΔCq method, with *Tubulin84* serving as the internal control gene. The qPCR analysis confirmed that *nlg3* transcript levels were significantly reduced in RNAi lines compared to controls. Efficacious knockdown was observed in both sexes, with females showing stronger reduction than males, as shown in Supplementary Fig. 3.

### Localizing Nlg3 protein using immunocytochemistry

#### Anti-Nlg3

Whole brains from 3- to 4-d-old males or females were fixed within 20 min of dissection in Bouin's solution (Fisher Scientific). Following fixation and washing, brains were blocked in 5% Normal Goat Serum in 1× PBS and stained with guinea pig anti-Nlg3 primary antibody (1:1,000, Invitrogen) overnight at 4 °C, which is a polyclonal primary antibody made and kindly provided by Dr. Brian Mozer (Yost et al. 2020). After washing, brains were stained with goat anti-guinea pig Alexa 488 (1:500, Invitrogen) for 2 h at room temperature. Next, all samples were left in glycerol for 1 h then mounted on slides using Fluoroshield media (Sigma-Aldrich), stored at 4 °C, and imaged the following day. Fluorescence was visualized with a AxioImager Z1 Compound Fluorescent microscope (Zeiss). Note: we used the entirety of our polyclonal antibody stock and have been unsuccessful in generating another functioning antibody for Nlg3. We switched to genetic constructs that are inserted in the *nlg3* gene and express GFP reporters under the regulatory elements of *nlg3* (*MiMIC-nlg3* and *nlg3-Gal4>mCD8-GFP*): both verify our findings.

#### Anti-GFP

Whole brains from 3- to 4-d-old males or females were fixed within 20 min of dissection in 4% paraformaldehyde in 1× PBS-T (0.3% Triton-X). Following fixation and washing, brains were blocked in 5% normal goat serum in PBS-T and stained with rabbit anti-GFP (1:1,000, Invitrogen) and mouse anti-Brp (nc82; 1:30, Developmental Studies Hybridoma Bank) for 48 h at 4 °C. After washing, brains were stained with Alexa 660 anti-mouse (1:400, Invitrogen) and Alexa 488 anti-rabbit (1:800, Invitrogen) for 48 h at 4 °C. Samples were mounted on slides in Fluoroshield media (Sigma-Aldrich), stored at 4 °C and imaged the following day. Slides were imaged on an SP8 Lightening Confocal microscopy system (Leica). All images were visualized with LAS X 2.0 software (Leica).

### Behavior assays

#### Social space assay

The assay was performed as previously described (McNeil et al. 2015; Yost et al. 2020). In short, flies were collected after eclosion and group housed until aged to 3 to 4 d old. Yost et al. (2024) have shown that most of the flies are no longer virgin at this age, matching the treatment conditions used for these experiments. Twenty-four hours prior to the assay, flies were sexed under cold anesthesia and separated into groups of 12 to 17 in vials with food for each treatment tested. Two hours before the assay, each treatment was transferred into new vials and acclimated to standard conditions (25 °C and 50% humidity). At the time of testing, flies were loaded into the triangular chamber and knocked to the bottom of the chamber to induce a startle response that quickly forces the flies into a group at the top of the chamber. Flies were then given 20 to 40 min to settle at their preferred social space. Each treatment was loaded into separate social space chambers and photos were taken as soon as flies had settled. For more details, see the video associated to the protocol description, in McNeil et al. (2015). Social space assay photos were processed with ImageJ (National Institute of Health, Bethesda, MA, USA) to calculate the average number of flies within a 4-body length radius (4BL or ∼1 cm) of each fly in the chamber using macros (available in Yost et al. 2020 and McNeil et al. 2015). An overview of the set up and the measurement of flies within 4BL is illustrated in Supplementary Fig. 4. Image analysis data were imported to GraphPad Prism 10 software for statistical analysis and graphical representation.

#### Climbing assay

The assay was performed as previously described (McNeil et al. 2015; Yost et al. 2020). Twenty-four hours before assaying, 2- to 4-d-old flies were sexed under cold anesthesia into groups of 40 for each treatment tested, and placed in vials containing food. Two hours before the assay, each treatment group was transferred into new vials and acclimated to testing conditions (25 °C and 50% humidity). Each treatment group was transferred into a testing tube and inserted into the climbing assay. Flies were forced to the bottom of the tube and given 15 s to climb to the tube on top of the apparatus. This short timeframe was chosen to allow approximately 80% of control flies to reach the top vial. After 15 s, the apparatus was closed and the number of flies in the top and bottom tubes were counted to calculate a performance index representing the percentage of flies that reached the top vial.

#### Temperature sensitive experiments

For these experiments, we used temperature-activated genetic constructs to compare the effect of altered neuronal transmission on social space. Temperature-sensitive treatment groups were assayed at 50% humidity and their respective activation temperatures: 27 °C for *TrpA1* flies to hyperactivate neurons, and 29 °C for *shi^ts^* flies to inhibit neurons. Simultaneously, control flies of the same genotype were assayed at 25 °C, so their neuronal transmission remained unaltered. Social spacing of all genetic background lines used to create the experimental lines was not affected by temperature changes (Supplementary Figs. 3 and 4), or in one case, an effect in the opposite direction from what was observed in the experiments (females MB/+ are sightly closer at 28 °C Fig. 3a, but further apart when used to drive *shi^ts^* or *TrpA1* Fig. 3b).

### Statistical analysis

Statistical analysis and graphical representation were performed in GraphPad Prism (RRID:SCR_002798, version 7.0a for Mac, GraphPad Software, La Jolla, CA, USA, www.graphpad.com). Prior to performing statistical tests, normality and homoscedasticity tests were used to assess whether the data followed a gaussian distribution, and the variance differed between the groups compared. When normality was confirmed, the appropriate 2-way ANOVA tests were performed, with data that had 2 independent variables and were collected simultaneously. Following the 2-way ANOVA, additional statistical tests were performed, Šídák's post-hoc tests, to assess the weight of the statistical evidence, especially relevant when interactions between the independent variables is found, while correcting for multiple comparisons (Supplementary Table 2). Supplementary Tables 3 and 4 report the ANOVA results and subsequent tests of the Supplementary figures. In the rare cases where the same controls were used for 2 experiments (Fig. 2a and b, as well as Supplementary Fig. 5a and b), and the *P*-values were <0.05, we added a *P*-value corrected for multiple comparisons in the corresponding tables, using the Bonferroni method (multiplying the *P*-value by the number of times the control was used for comparison, in all cases it was 2) Those corrections did not affect the interpretations. *P* values are reported on the figures when there is statistical support for evidence of effect (Greenland et al. 2016).

### Drawings

Drawings presented in the main text figures were created on the app Procreate 5.3.5 (Savage Interactive Pty Ltd, Hobart, Australia).

## Results

### The experimental lines showed sufficient locomotor ability to be assessed in the social space assay

We first used climbing assays to confirm that all lines could reach the top of the social space chamber. This assay tests stamina, startle response, and locomotion. Any fly line with individuals reaching the top vial within 15 s (performance index, PI > 0) can be tested in the social space assays (McNeil et al. 2015; Yost et al. 2020). Because flies have at least 20 min to settle in the social space chamber before pictures are taken, this criterion is sufficient to rule out major movements confounds. All lines met it. Climbing data are provided in Supplementary Figs. 4–8. As previously reported (Jiang et al. 2020; Yost et al. 2024), we find no association between changes in locomotion deficits and changes in social space (Supplementary Tables 5 and 6).

### Neuroligin 3 protein is expressed at low levels in the calyx of the mushroom bodies, the protocerebral bridge, and the optic lobes

We examined Nlg3 protein expression in the adult brain with an anti-Nlg3 polyclonal antibody. Nlg3 protein localized at low levels to the calyx (Ca) of the mushroom bodies (MB), the protocerebral bridge (PB), and the optic lobes (OL) against lipid autofluorescence background (Fig. 1a and b). We confirmed this pattern with 2 *nlg3* reporter lines: a *MiMIC* p-element insertion within *nlg3*, and a *nlg3-Gal4* insertion (see Supplementary Table 1). The *MiMIC-nlg3* insertion incorporates a *EGFP* reporter into the coding sequence of the host gene *nlg3* (Venken et al. 2011). It showed low GFP expression in the MB and OL and at even a lower level in the PB (Fig. 1c and d). The *nlg3-Gal4* insertion driving *mCD8-GFP* confirmed expression at higher levels than endogenous *nlg3* (Fig. 1e and f). Male and female localization of Nlg3 did not appear to be different qualitatively or quantitatively in 3- to 4-d-old adult flies (representative images of only the males are shown).

**Fig. 1.**
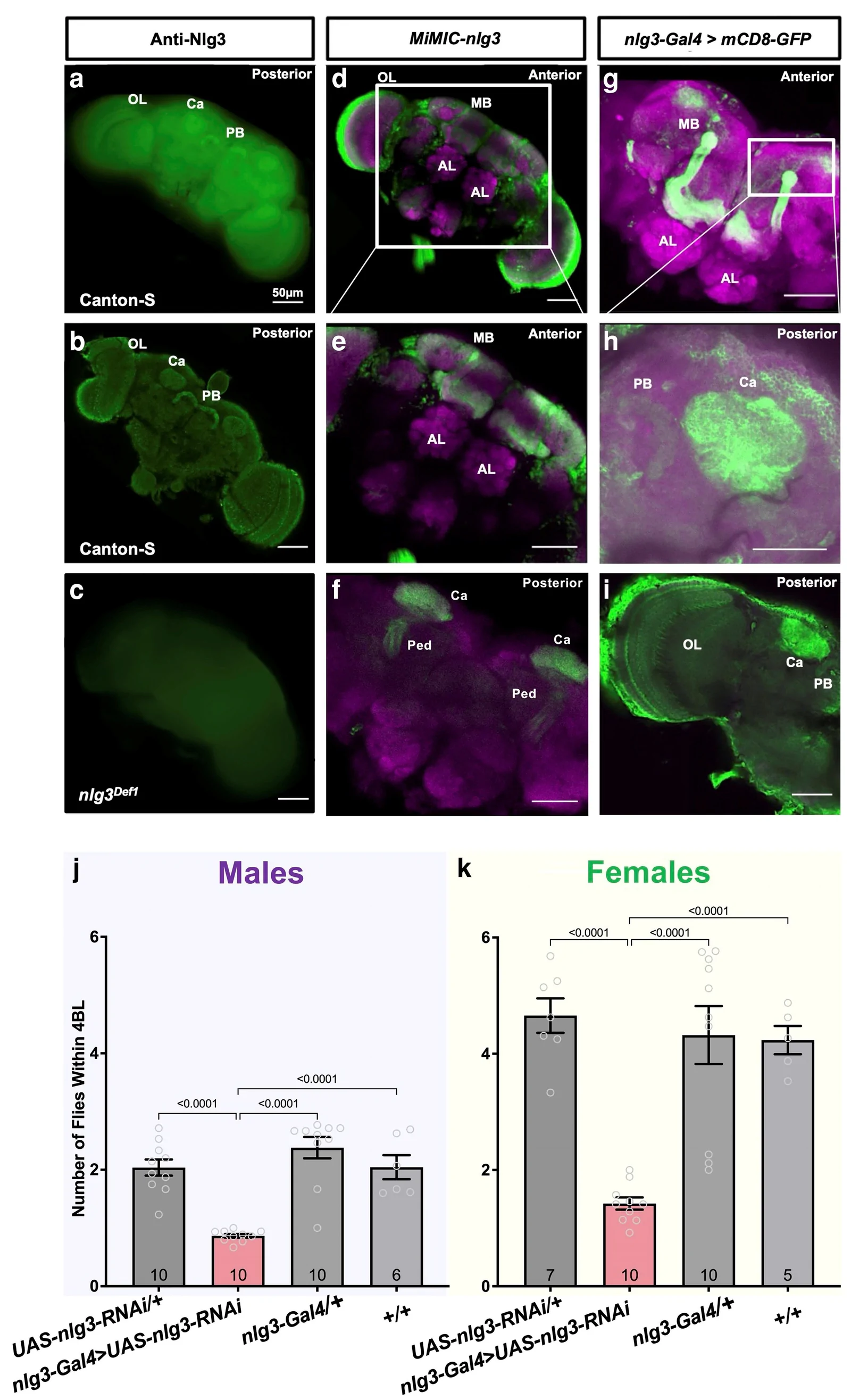
Nlg3 is expressed in the calyx of the mushroom bodies, throughout the protocerebral bridge, and in the optic lobes, and that expression is important for social space. a–c) Immunofluorescent imaging of polyclonal antibody specific to the c-terminal intracellular domain with anti-Nlg3 (green) in Canton-S (a, b) and *nlg3^Def1^* (c) male fly whole brains dissected at 3 to 4 d old. Magnification: 20×. a) Fluorescent imaging on posterior side of adult brain shows 3 main structures expressing Nlg3 in low levels: calyx of the MB (Ca), protocerebral bridge (PB), and the optic lobes (OL). b) Confocal imaging on posterior side of adult brain shows definition of Nlg3 expression in the Ca, PB, and OL (medulla and lamina). c) Fluorescent imaging on the posterior side of a complete *nlg3* deletion mutant adult brain has no localized enrichment. d–f) Expression patterns of *MiMIC* construct (*Mi{y[+mDint2] = MIC}nlg3[MI08924]*) incorporating *EGFP* into *nlg3*. Immunofluorescence with anti-GFP (green) and nc82 (magenta). d) Confocal imaging on anterior side of adult brain at 20× magnification indicates low expression of *nlg3* in the MB and OL. e) Confocal imaging at 40× magnification highlighting expression in the MB. f) Confocal imaging at 40× magnification of the posterior side highlighting expression in the Ca and pedunculus (Ped). g–i) Expression patterns of *MiMIC nlg3-Gal4* genetic construct (*Mi{Trojan-Gal4}nlg3>UAS-mCD8::GFP*) driving exogenous expression of *membrane-bound-GFP*. Immunofluorescence with anti-GFP (green) and nc82 (magenta). g) Confocal imaging of posterior side of adult brain at 40× magnification indicates low expression patterns in PB and highlights the Ca. h) Confocal imaging of anterior side of adult brain at 40× magnification shows expression throughout the MB. Antennal lobes (AL) shown for reference. i) Confocal imaging of the posterior side of adult brain at 40× magnification shows expression through the medulla and lamina layers of the OL. High fluorescence is also seen in the Ca with lower expression also in the PB. Scale bar, 50 µm. j,k) Social space of flies with RNAi knockdown of *nlg3* targeted specifically to all *nlg3*-expressing cells. There is a decrease in the average number of flies within 4 body lengths (4BL) in male (J; one-way ANOVA*: F*_3,32_ = 1.771, *P* = 0.0003, post-hoc Šidák's test *P* < 0.0001 when comparing *nlg3*>UAS-RNAi *nlg3* to all controls) and female (K; 1-way ANOVA*: F*_3,28_ = 3.967, *P* < 0.0001, post-hoc Šidák's test *P* < 0.0001 when comparing *nlg3*>UAS-RNAi *nlg3* to all controls).

Next, we used the *nlg3-Gal4* driver to express an RNAi specific to *nlg3* (outcrossed in the Cs background) in all cells that express *nlg3* (Fig. 1g and h). These results phenocopy previously reported observations on flies deficient for the *nlg3* gene, and mutations in the same gene also display disrupted social spacing (Yost et al. 2020, 2024). Both male and female *nlg3-Gal4*>*UAS-nlg3 RNAi* flies displayed a decrease in the number of flies within 4BL (increased social space) compared to their genetic control fly lines (*nlg3*-Gal4*/+* and *RNAi*/+, all outcrossed in Cs genetic background—One-Way ANOVA *P* < 0.0001, post-hoc comparison of *nlg3-Gal4*>*UAS-nlg3 RNAi* to other controls *P* < 0.0001, Fig. 1g and h).

### Neuroligin 3 expression in the mushroom bodies, protocerebral bridge, and the optic lobes is required for normal social spacing in both sexes

To determine the behavioral impact of Nlg3 expression in each brain structure, we targeted RNAi to knock down *nlg3* expression independently in each of the MB, PB, and OL. In all structures, independently, we recapitulated (with a smaller effect size) the increase in social space observed with the *nlg3-Gal4* driving *nlg3*-RNAi: for the MB (Fig. 2a; 2-way ANOVA—Effect of genotype: *F*_1,39_ = 14.71, *P* = 0.0004), the OL (Fig. 2c; 2-way ANOVA—Effect of genotype: *F*_1,31_ = 7.191, *P* = 0.0116, no interaction between sex and genotype, effect mostly driven by females, post hoc Šídák’ test comparing females *P* = 0.032), and in the PB (Fig. 2b; 2-way ANOVA—Effect of genotype: *F*_1,40_ = 19.02, *P* < 0.0001, with interaction between sex and genotype: *F*_1,40_ = 5.607, *P* < 0.0228, driven by the females, post hoc Šídák’ test comparing females *P* < 0.0001).

**Fig. 2.**
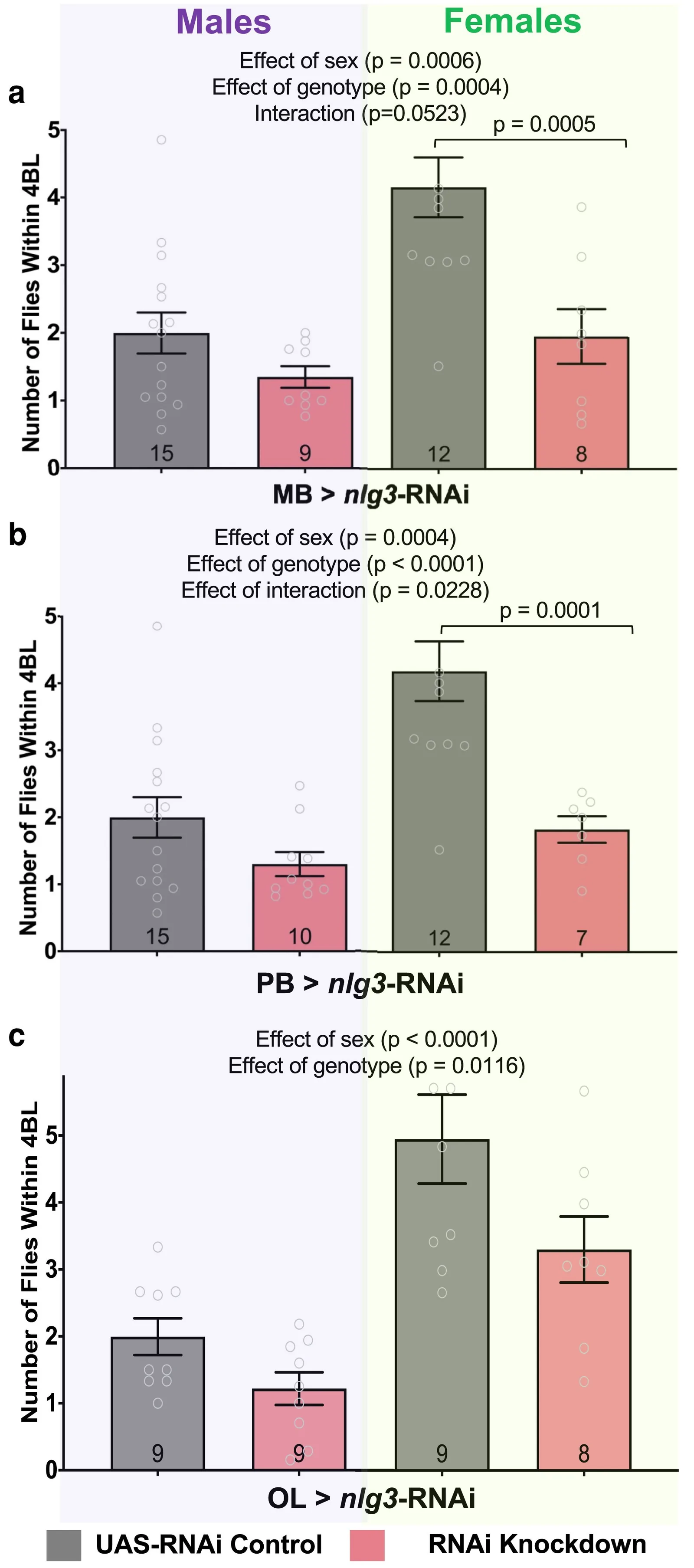
Knocking down Nlg3 specifically in each of the mushroom bodies, the protocerebral bridge, or the optic lobes increases fly social space in males and females. a–c) Average number of flies within 4 body lengths (4BL) in male and female flies with RNAi knockdown of *nlg3* targeted specifically to the mushroom bodies (a), protocerebral bridge (b), and the optic lobes (c). a) The average number of flies within 4BL decreased in both males and females in flies with *nlg3* knockdown targeted to the MB (2-way ANOVA—Effect of genotype*: F*_1,39_ = 14.71, *P* = 0.0004). b) The average number of flies within 4BL decreased in female flies with *nlg3* knockdown targeted to the PB (2-way ANOVA—Effect of genotype: *F*_1,40_ = 19.02, *P* < 0.0001, with interaction between sex and genotype: *F*_1,40_ = 5.607, *P* < 0.0228, driven mostly by the females, post hoc Šídák’ test comparing females *P* = 0.0001). c) Knocking down *nlg3* in the OL decreased the average number of flies within 4BL in both males and females compared to genetic control flies (2-way ANOVA—Effect of genotype: *F*_1,31_ = 7.191, *P* = 0.0116). Left side, in gray color: genetic control *RNAi/+*; right side, in pink color: *driver > RNAi.* Error bars are ±s.e.m., *n* = 7–14 for all treatments, results of Šídák's post hoc are shown above the bars when they strongly support the differences observed. The results of all 2-way ANOVA and *post-hoc* tests performed can be found in Supplementary Table 2.

### Hyperactivating and silencing nlg3-expressing neurons, independently or simultaneously, changes fly social space in a sex-specific manner

Next, using genetic tools, we manipulated neuronal transmission within the MB, PB, and nlg3-neurons. When hyperactivating all neurons within the MB, we saw a lower number of flies within 4BL, indicating increased social space in both sexes (Fig. 3a; 2-way ANOVA—Effect of neuronal transmission: *F*_1,47_ = 7.395, *P* = 0.0091). Silencing all MB neurons also increased social space in both males and females (Fig. 3a; 2-way ANOVA—Effect of neuronal transmission: *F_1,30_* = 14.40, *P* = 0.0007). In contrast, hyperactivating PB neurons did not change fly social space, though the females displayed a trend for higher number of flies within 4BL (decreased social space). Silencing PB neurons in males had no effect, but in females’ social space was increased (Fig. 3b; 2-way ANOVA—Effect of neuronal transmission: *F*_1,31_ = 3.443*, P* = 0.0730; post hoc Šídák’ test comparing females *P* = 0.0596). Finally, we used the *nlg3-Gal4* driver to manipulate the transmission of all *nlg3*-expressing neurons. Hyperactivating all *nlg3*-expressing neurons increased social space in both males and females (Fig. 3c; 2-way ANOVA—Effect of neuronal transmission: *F*_1,28_ = 12.70, *P* = 0.0013). Silencing these neurons increased social space in females but not males (Fig. 3c; 2-way ANOVA—Effect of interaction: *F*_1,22_ = 6.169, *P* = 0.0211, post hoc Šídák’ test comparing females *P* = 0.0042).

**Fig. 3.**
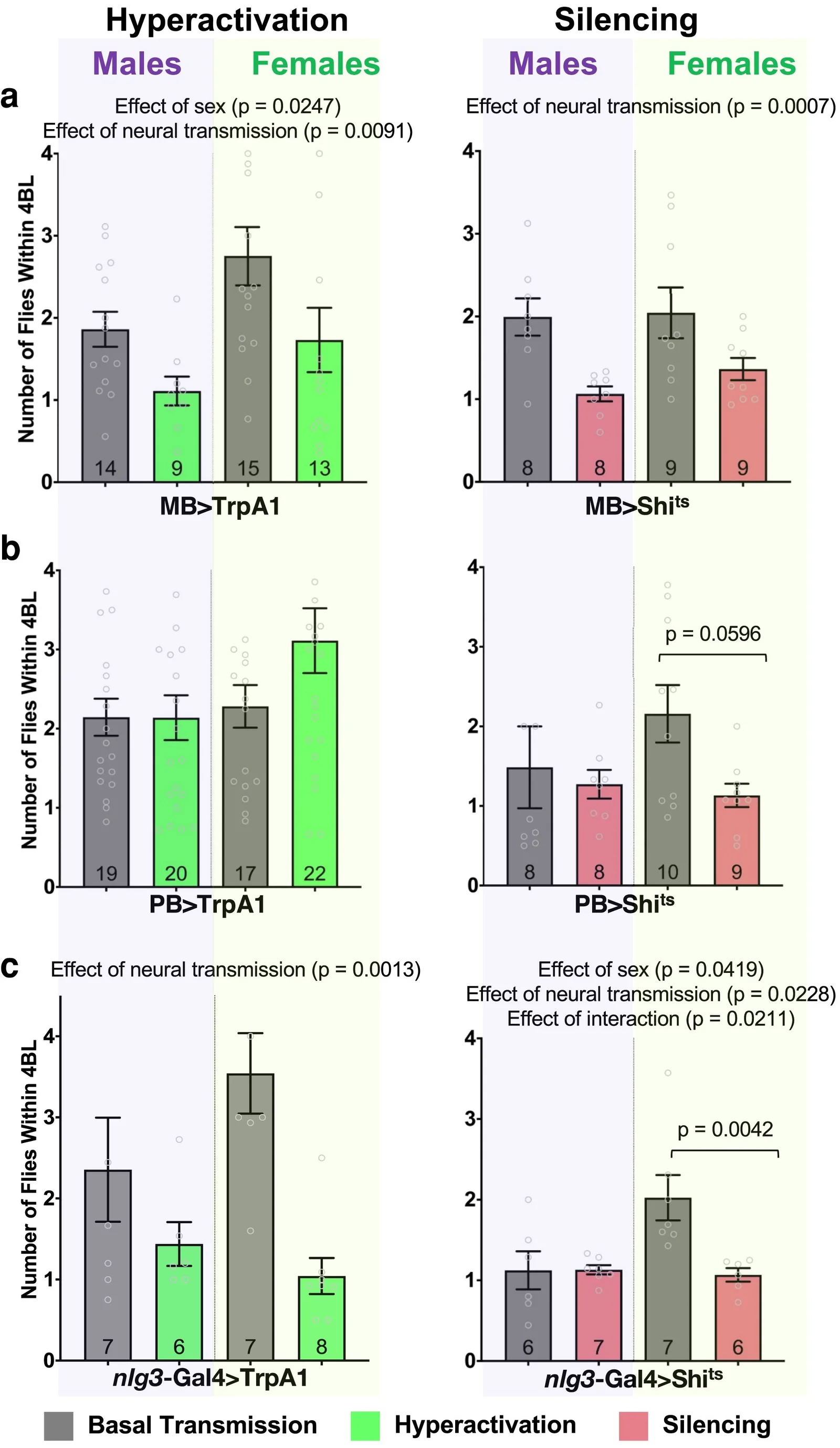
Manipulation of neuronal transmission within the brain structures enriched with Nlg3 increases fly social space. a—c) Average number of flies within 4 body lengths (4BL) in male and female flies when genetically hyperactivating or silencing neural transmission within the MB (a), PB (b), and *nlg3*-containing neurons (c). a) Hyperactivation of MB neurons decreases the number of flies within 4BL in both males and females compared to basal transmission of MB neurons (2-way ANOVA—Effect of neuronal transmission: *F*_1,47_ = 7.395, *P* = 0.0091). Silencing MB neurons also decreases the average number of flies within 4BL in both males and females compared to basal transmission (2-way ANOVA—Effect of neuronal transmission: *F*_1,30_ = 14.40, *P* = 0.0007). b) The average number of flies within 4BL was not different in males and females when the PB neurons are hyperactivated (2-way ANOVA—Effect of neuronal transmission: *F*_1,74_ = 17.72, *P* = 0.2018) or silenced (2-way ANOVA—Effect of neuronal transmission: *F*_1,31_ = 3.443, *P* = 0.0730). c) Hyperactivation of *nlg3-*containing neurons decreases the average number of flies within 4BL in both males and females compared to basal transmission (2-way ANOVA—Effect of neuronal transmission: *F*_1,28_ = 12.70, *P* = 0.0013). There is an interaction between sex and silencing *nlg3*-containing neurons where females have a decrease in the average number of flies within 4BL (2-way ANOVA—Effect of interaction: *F*_1,22_ = 6.169, *P* = 0.0211, post hoc Šídák’ test comparing females *P* = 0.0042). Left side, gray color bars: inactivated genetic construct and basal transmission of neurons; right side in green color bars, under Hyperactivation heading: activation of genetic construct and hyperactivation of neurons with *Gal4* driver; left side, in pink color bars, under Silencing heading: activation of genetic construct and silencing of neurons with *Gal4* driver. Error bars are ±s.e.m., *n* = 6–22 for all treatments. The results of all 2-way ANOVA and *post-hoc* tests performed can be found in Supplementary Table 2.

### Sexually dimorphic neurotransmitter involvement in the MB

As the MB are important for fly's social space, we investigated how the main (and probably only) neurotransmitter released by the MB, acetylcholine, may be underlying the observed effects on social space. Using UAS-RNAi, we knocked down rate-limiting biosynthesis enzyme within the MB, namely *choline acetyltransferase (ChAT* for acetylcholine production) (Shih et al. 2019). Acetylcholine reduction in the MB decreased fly social space in males and females, but with a larger effect size in females (Fig. 4a; 2-way ANOVA—Effect of genotype: *F*_1,32_ = 11.19, *P* = 0.0021; post hoc Šídák’ test comparing females *P* = 0.0022).

**Fig. 4.**
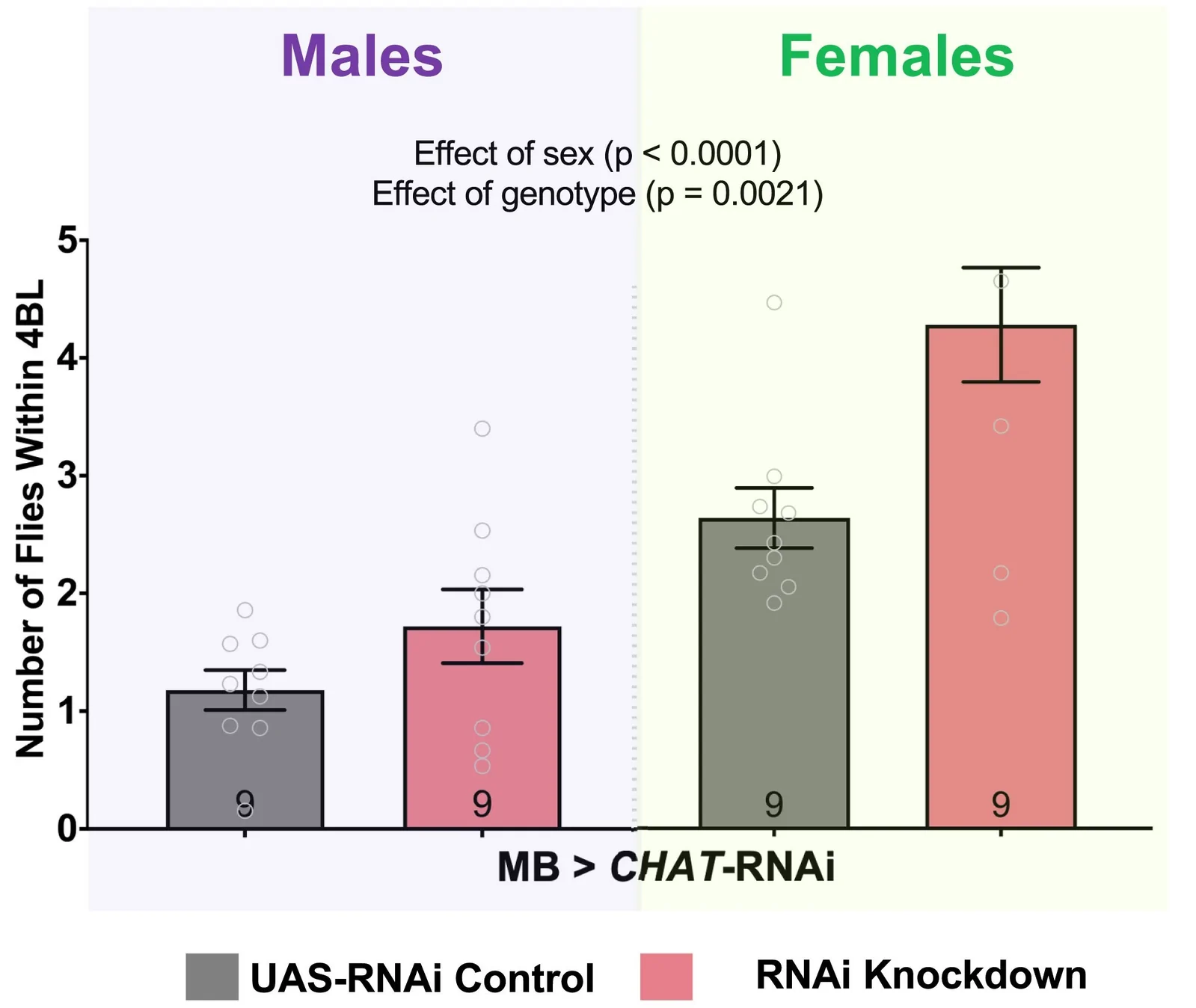
Knocking down the acetylcholine rate-limiting biosynthesis enzyme C*hAT* in the MB decreases fly social space in both sexes. Average number of flies within 4 body lengths (4BL) in male and female flies with RNAi knockdown of *ChAT* for acetylcholine synthesis specifically in the mushroom bodies. Both male and female flies have an increased average number of flies within 4BL with *ChAT* knockdown when compared to the genetic control flies (2-way ANOVA—Effect of genotype: *F*_1,32_ = 11.19, *P* = 0.0021). Under each male or Female headings, on the left side, in gray color bars: genetic control *RNAi/+*; on the right side, in pink color bars: *driver>RNAi*. Error bars are ±s.e.m., *n* = 8–9 for all treatments, results of Šídák's post hoc are shown above the bars when they strongly support the differences observed. The results of all 2-way ANOVA and *post-hoc* tests performed can be found in Supplementary Table 2.

Previous research shows global dopaminergic changes in the adult fly brain affect social space (Fernandez et al. 2017; Xie et al. 2018; Yost et al. 2024). However, the anatomical locations within the adult fly brain where alterations in dopaminergic transmission affect social space is undefined. Thus, we tested the dopaminergic inputs into the MB, and found that manipulating the expression of each dopamine receptor within the MB increased social space, with a larger effect size in males than females: *Dop1R1* (Fig. 5a; 2-way ANOVA—Effect of genotype: *F_1,32_* = 68.23, *P* < 0.0001), Dop1R2 (Fig. 5b; 2-way ANOVA—Effect of genotype: *F*_1,32_ = 13.19, *P* = 0.001), Dop2R (Fig. 5c; 2-way ANOVA—Effect of genotype: *F*_1,32_ = 41.10, *P* < 0.0001), and DopEcR (Fig. 5d; 2-way ANOVA—Effect of genotype: *F*_1,32_ = 8.905, *P* = 0.0054).

**Fig. 5.**
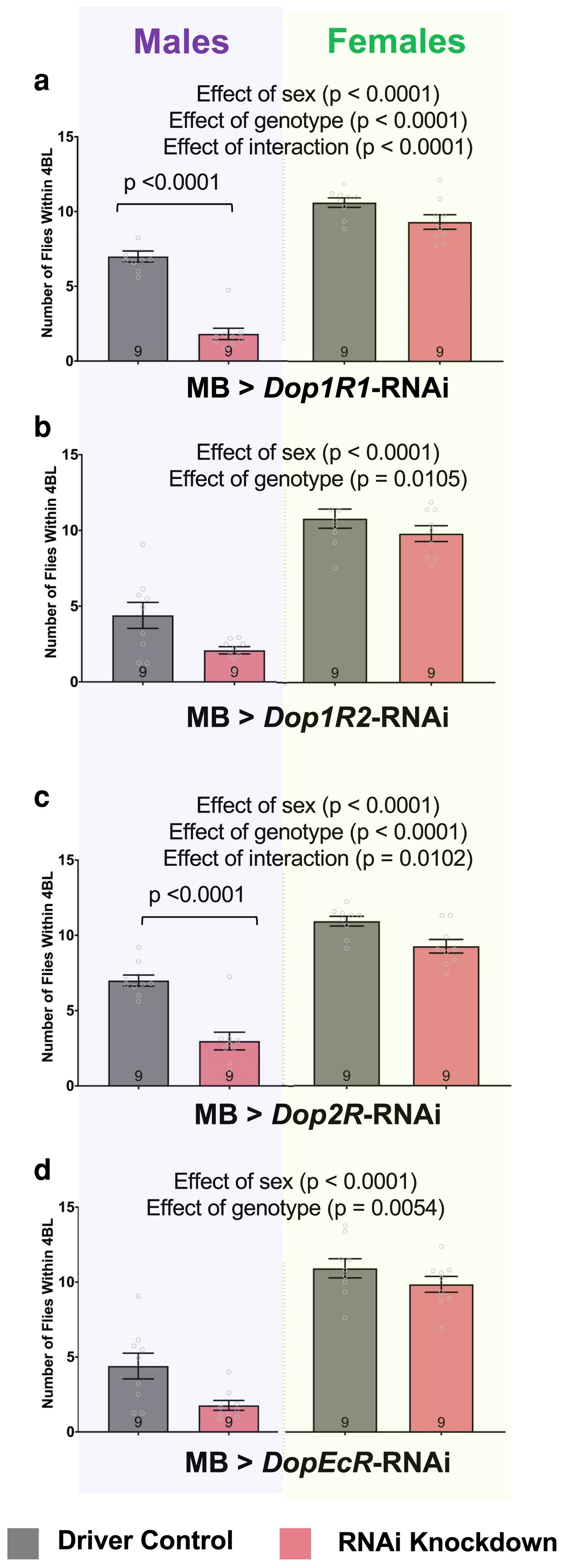
RNAi manipulation of all dopamine receptors in the MB increases social space in males. a–d) Average number of flies within 4 body lengths (4BL) in male and female flies RNAi manipulations of dopamine receptors *Dop1R1* (a), *Dop1R2* (b), *Dop2R* (c), and *DopEcR* (d) specifically in the mushroom bodies. All receptors’ knockdowns in the MB result in a decreased average number of flies within 4BL compared to genetic controls, with a large effect size in males, and a small to no effect in females. a) Dop1R1 (2-way ANOVA—Effect of genotype: *F*_1,32_ = 68.23, *P* < 0.0001). b) Dop1R2 (2-way ANOVA—Effect of genotype: *F*_1,32_ = 7.401, *P* = 0.0105). c) Dop2R (2-way ANOVA—Effect of genotype: *F*_1,32_ = 41.10, *P* < 0.0001). d) DopEcR (2-way ANOVA—Effect of genotype: *F*_1,32_ = 8.905, *P* = 0.0054). Under each of Males or Females headings, on the left side, in gray color bars: genetic control *driver/+*; on the right side, in pink color bars: *driver>RNAi*. Error bars are ±s.e.m., *n* = 9 for all treatments, results of Šídák's post hoc are shown above the bars when they reach strong support of evidence. The results of all 2-way ANOVA and *post-hoc* tests performed can be found in Supplementary Table 2.

### Neurons expressing the fruitless gene under the P1 promoter modulate sexually dimorphic social spacing responses

Due to the observed sex differences in the neural substrates underlying social spacing preferences, we looked at a subset of sexually dimorphic *fruitless*-expressing neurons: those expressing *fruP1*. While hyperactivating *fruP1* neurons increased social space for both sexes (Fig. 6; 2-way ANOVA—Effect of neuronal transmission: *F*_1,33_ = 22.65, *P* < 0.0001), silencing *fruP1* neurons resulted in a sex-specific effect with a decrease in social space for males (Fig. 6; 2-way ANOVA and Šídák's *post-hoc* test: *F*_1,30_ = 3.451, *P* = 0.0453) and no effect on female social space.

**Fig. 6.**
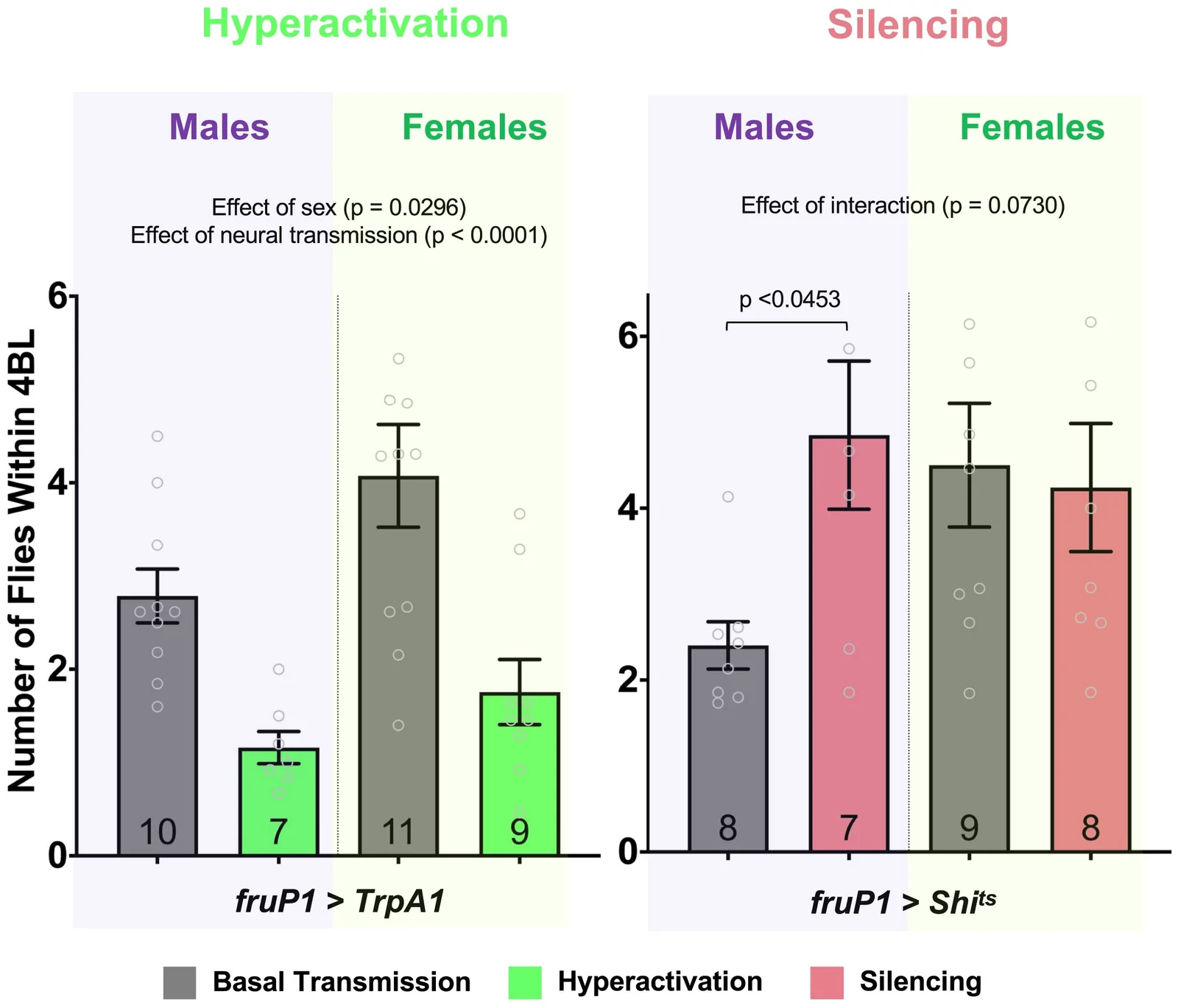
Hyperactivation of f*ruP1* neurons increases social space in males and females while silencing *fruP1* neurons only decreases social space in males. Average number of flies within 4 body lengths (4BL) in males and females when genetically hyperactivating neuronal transmission in *fruP1* neurons. Hyperactivating *fruP1* neurons decreases the average number of flies within 4BL in both males and females compared their basal transmission (2-way ANOVA—Effect of neuronal transmission: *F*_1,33_ = 22.65, *P* < 0.0001). Silencing *fruP1* neurons did not alter female social space but decreased the average number of flies within 4BL in males (2-way ANOVA with Šídák's *post-hoc* test: *P* = 0.0453). Under each heading, on the left, in gray color bars: inactivated genetic construct and basal transmission of neurons; under Hyperactivation heading, on the right side, in green color bars: activation of genetic construct and hyperactivation of neurons with Gal4 driver; under Silencing heading, on the right, in pink color bars: activation of genetic construct and silencing of neurons with Gal4 driver. Error bars are ±s.e.m., *n* = 7–11 for all treatments. The results of all 2-way ANOVA and *post-hoc* tests performed can be found in Supplementary Table 2.

To assess if *nlg3* within *fruP1*-expressing neurons affects social space, we assayed flies with *UAS-nlg3 RNAi* driven by the *fruP1* driver. Reducing levels of *nlg3* in *fruP1* neurons resulted in decreased social space in females, but no effect in males (Fig. 7a; 2-way ANOVA—interaction between sex and genotype *F*_1,57_ = 4.09, *P* = 0.0478; Šídák's post hoc comparing the genotypes in females *P* < 0.0001). Next, we investigated the neurotransmitters involved in fly social space in *fruP1* neurons. Knocking down *TH*, the rate limiting-enzyme in the dopamine synthesis pathway, in *fruP1* neurons did not affect male or female social space (Fig. 7b; 2-way ANOVA—Effect of genotype: *F*_1,329_ = 0.413, *P* = 0.5255). In contrast, reducing acetylcholine levels by knocking down *ChAT* in *fruP1* neurons decreased social space in both males and females (Fig. 7c; 2-way ANOVA—Effect of genotype: *F*_1,48_ = 99.11, *P* < 0.0001). Similarly, reducing GABA by knocking down *GAD1* in *fruP1* neurons decreased male and female social space (Fig. 7d; 2-way ANOVA—Effect of genotype: *F*_1,42_ = 8.478, *P* = 0.0057).

**Fig. 7.**
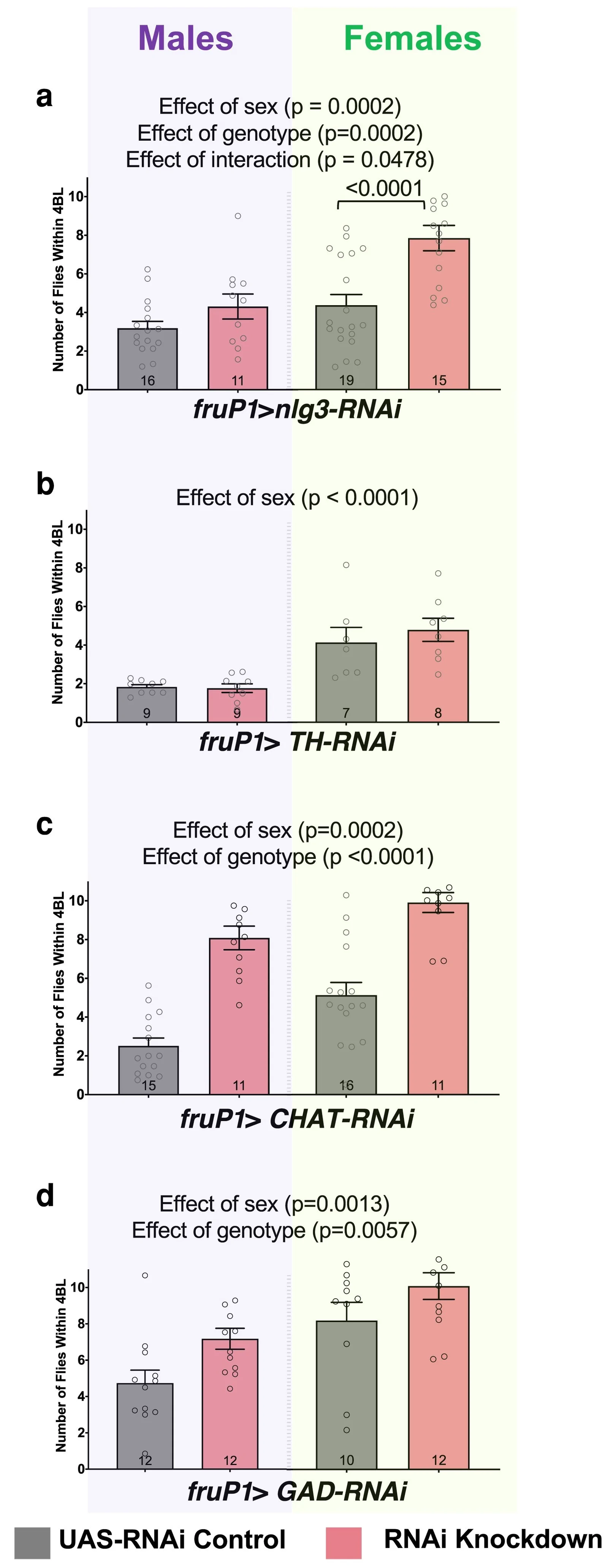
Knocking down *nlg3* in *fruP1* neurons decreases only females’ social space, while a reduction of acetylcholine and GABA in *fruP1* neurons decrease social space in both sexes. a–d) Average number of flies within 4 body lengths (4BL) in male and female flies with RNAi knockdown of *nlg3* (a), *TH* for dopamine synthesis (b), *ChAT* for acetylcholine synthesis (c) or *GAD1* for GABA synthesis (d) specifically in *fruP1* neurons. a) Knocking down *nlg3* in *fruP1* increases the average number of flies within 4BL in females, with no effect in males (2-way ANOVA—interaction between sex and genotype *F*_1,57_ = 4.09, *P* = 0.0478; Šídák's post hoc comparing the genotypes in females *P* < 0.0001). b) *TH* knockdown in *fruP1* neurons does not change social space in either sex (2-way ANOVA—Effect of genotype: *F*_1,29_ = 0.413, *P* = 0.5255). c) *ChAT* knockdown in *fruP1* neurons increases the average number of flies within 4BL in both males and females (2-way ANOVA—Effect of genotype: *F*_1,47_ = 99.11, *P* < 0.0001). d) Similarly, *GAD1* knockdown in *fruP1* neurons increases the average number of flies within 4BL in both males and females (2-way ANOVA—Effect of genotype: *F*_1,42_ = 8.478, *P* = 0.0057). Under each of the Males and Females heading, on the left, in gray color bars: genetic control *RNAi/+*; on the right, in pink color bars: *driver>RNAi*. Error bars are ±s.e.m., *N* = 9–13 for all treatments. The results of all 2-way ANOVA and *post-hoc* tests performed can be found in Supplementary Table 2.

## Discussion

Social behaviors, including social space, require a neuronal integration of perceived attractive and repulsive cues from other individuals. Here, we expand on mapping the neuronal basis of social behavior by focusing on the sexually dimorphic role of *neuroligin 3* and its associated adult brain structures in *Drosophila melanogaster* social spacing.

The fact that we and others report no correlation between deficits in locomotion and preferred social space may be counter intuitive. However, Jiang et al. (2020) report in their detailed description of fly cluster formation that locomotion deficits do not underlie deficits in social clustering. We have shown here, and before (McNeil et al. 2015; Yost et al. 2020) that moderate decreased climbing performances do not predict social spacing preferences. Nevertheless, fine motor differences could alter how flies perceive each other and adjust their preferred social space. Further analysis, using other behavior assays, would address this issue.

Across most genotypes, females displayed closer social spacing than males (2-way ANOVA: effect of sex, *P* < 0.05), this aligns with previously published results using our genetic background fly line, Cs (Simon et al. 2012; Yost et al. 2020, 2024), although some other backgrounds did not show such difference (Jiménez-Padilla et al. 2024). Since the effect of genetic background on the different preferred social space of males vs females is known, and independent from the fact that males and females respond differently to the same manipulations, the following sections focus on genotype and interaction effects.

We show for the first time that the Nlg3 protein shows an enrichment in its expression in the mushroom bodies, optic lobes, and protocerebral bridge in the adult fly brain. These structures independently require *nlg3* for normal social spacing in both sexes (with a stronger effect size apparent in females). The importance of Nlg3 in the OL on social behavior was expected as vision is known to be important for proper social spacing (Simon et al. 2012; Burg et al. 2013; Jiang et al. 2020). The involvement of both the OL and MB further supports the idea that social spacing requires integration of sensory perception with higher-order behavioral decision-making.

In flies with *nlg3* knockdown, we recapitulate the reported social spacing phenotypes seen in flies deficient of the full *nlg3* gene. However, here, younger females also show a change in social space, whereas *nlg3*-deficiency produced an effect of age in females (Yost et al. 2020, 2024). Insertion mutations or overexpression of *nlg3* also disrupts social spacing, although in a sex-specific manner (Yost et al. 2020, 2024), indicating that the large introns in which those insertions are located probably contain sex-specific regulatory elements. We also note that the first intron of the *nlg3* gene contains a long non-coding RNA:CR44332, in the same direction as the gene, and of unknown function (based on Flybase JBrowse—Öztürk-Çolak et al. 2024). That long non-coding RNA does not align with any sequence other than the intron itself, which suggests that CR44332 could be involved in transcriptional regulatory activities of *nlg3*. (Soutschek and Schratt 2023; He and Kang 2024). It is possible that the discrepancy between our observation with a deletion of the DNA sequence vs an alteration of the mRNA transcript function could be related to the loss of sex-specific regulatory elements, including CR44332. Additional studies will be necessary in the future to assess the possible role of the long non-coding RNA:CR44332 in the phenotypes we observe, as well as the possibility of sex-specific regulatory elements in the introns.

Additionally, comparing the effect of total neuron transmission manipulations in each brain structure highlighted the importance of the MB in regulating social space. Hyperactivating and silencing MB neurons in both males and females increased social space. However, hyperactivating and silencing the PB only affected social space in females, with a reciprocal effect: hyperactivating PB neurons led to the females tending to be closer, while silencing those neurons led to the flies being further apart. Using the *nlg3*-Gal4 driver we reproduced some of the individual PB and MB effects: hyperactivating all *nlg3* neurons increased social space in both sexes, as seen when hyperactivating or silencing the MB, while silencing all *nlg3* neurons resembled the effect of PB silencing where only females were affected, and further apart.

In contrast to the lack of sexual dimorphism observed when manipulating total neuron transmission in the MB, dopaminergic inputs to the MB mostly change males’ social space. These findings suggest that the circuitry underlying social space through the MB is in fact sexually dimorphic, and we found that acetylcholine release from the MB more strongly modulates female social space than males. We still do not know the specific inputs to the MB leading to changes in females. Together, these findings suggest that males and females can display similar alterations in social spacing while relying on partially distinct neural substrates and neurotransmitter systems.

We showed that hyperactivating *fruP1* neurons affected social space in a sexually dimorphic manner, as did silencing them. This demonstrates the influence *fru* neurons have on social space along with other sexually dimorphic social behaviors in flies, such as male courtship (Sato et al. 2019) or female receptivity (Chowdhury et al. 2020).

Finally, manipulating neurotransmitters within *fruP1* neurons revealed that dopamine release from those neurons was not important for social space. Instead, both acetylcholine and GABA release from *fruP1* neurons play a role in fly social space, a phenocopying the effect of acetylcholine knockdown in the MB. In addition, RNAi knockdown of *nlg3* in the *fruP1* neurons only affected female social spacing. Altogether those results led us to propose a social space circuitry model in Fig. 8.

**Fig. 8.**
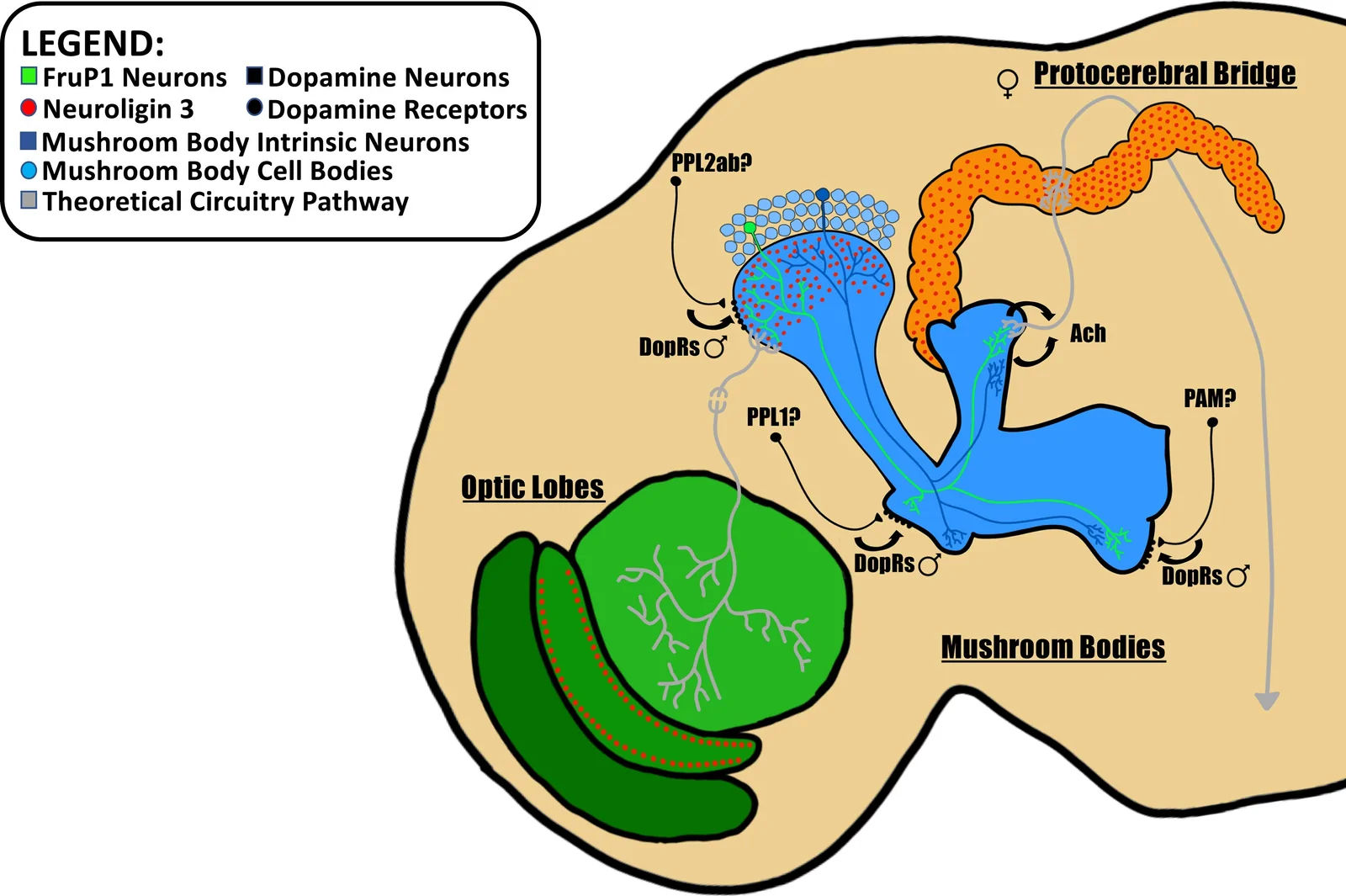
Proposed theoretical model of functional neural influences on Drosophila social space. Dopaminergic inputs into the mushroom body neurons (dark blue line—sex text) influence male social space while cholinergic outputs from the mushroom bodies influence female social space. Cholinergic outputs from *fruP1* neurons (light green line) also influence male and female social space while some mushroom body neurons also express *fru*. Neuroligin protein (red dots) enrichment is also found in the optic lobes, calyx of the mushroom bodies, and protocerebral bridge. Of note, not shown, there could be a direct input of *nlg3* neurons directly into the PB.

### *Nlg3* localization in mushroom bodies, protocerebral bridge, and optic lobes

Our antibody targeted the intracellular domain of Nlg3, which allowed us to identify protein localization specifically in the calyx of the MB (containing the dendritic ends of the Kenyon cells), the medulla and lamina layers of the OLs, and throughout the entire PB (Fig. 1a and b). We also confirmed a low level of *nlg3* expression in the adult 3- to 4-d-old brain, which was previously observed by Yost et al. (2020), using Western blots and mRNA quantification.

Attempts to create another polyclonal, or even a monoclonal antibody specific to Nlg3 were unsuccessful, so we transitioned to using 2 different genetic constructs (*MiMIC-nlg3* and *nlg3-Gal4>mCD8-GFP*) to confirm the results we observed with our polyclonal antibody. Similar to the Nlg3 localization visualized using the polyclonal antibody, GFP localized to the MB and OL in *MiMIC-nlg3* flies (Fig. 1c and d). However, the GFP expression in the *MiMIC-nlg3* flies was also low, and we could not see any expression in the PB. This was probably due to the endogenously low expression of *nlg3* as well as the fact that EGFP would be cytoplasmic in this transgenic construct. We then switched to a *nlg3-Gal4* driver, and a membrane-bound GFP (Fig. 1f) leading to a better visualization with immunofluorescence of the OL, PB and the calyx of the MB (Fig. 1e).

### Importance of *nlg3* in each of: the mushroom bodies, protocerebral bridge, and optic lobes in social spacing

Deletion of the whole *nlg3* gene leads to an increase in fly social space (Yost et al. 2020), which we replicated with a *nlg3-*RNAi knockdown using the MB, PB, OL, and *nlg3-Gal4* drivers. However, females displayed a stronger effect size in our experiments, which could be due to the difference in efficacy of the RNAi knockdown in females vs males (see Supplementary Fig. 2), although each driver may cause sex-specific levels of expression. Finally, we cannot exclude that the role of *nlg3* in those structures could be sex-specific.

### Importance of neuronal activity in each of MB and PB for social spacing

We then assessed the potential roles of the MB and PB in social space, not through manipulations of *nlg3*, but by modifying transmission of their neurons. We also observed an increase in male and female social space whether the MB neurons were hyperactivated or silenced (Fig. 3a). The MB is not only important for learning and memory, but also sensory integration; it would follow that manipulation of these neurons would affect social behavior.

While manipulating PB neuron transmission did not have an effect in males, there is a reciprocal trend in females, where hyperactivating them decreases social space, and silencing increases social space (Fig. 3b), indicating a sex-specific role for the PB in regulating this behavior in females.

A sexually dimorphic effect was observed when we manipulated the transmission of all *nlg3*-expressing neurons: females were further apart whether these neurons were hyperactivated or silenced, and males were further apart with hyperactivation but not silencing of the same neurons (Fig. 3c). This sexual dimorphism in how neuronal activity affects social spacing might explain the sexually dimorphic effect of the *nlg3* deletion reported by Yost et al. (2020). These results strongly suggest that the different structures expressing *nlg3* interact with each other to affect social space, and that each structure might play a different role depending on sex.

### Neurotransmitters in the MB that modulate social space: dopamine reception in males, and acetylcholine release in both sexes

While the main MB inputs entering at the calyx and received by the Kenyon cells are GABA and acetylcholine, and potentially glutamate (Schürmann, 2000; Yasuyama et al. 2002; Ganguly et al. 2024), the Kenyon cells release the neurotransmitter acetylcholine (Barnstedt et al.; 2016), and transcriptomics studies found no evidence of other small neurotransmitters (Shih et al. 2019). However, a neuromodulator is also released by the Kenyon cells: the short neuropeptide F (sNPF—Knapek et al. 2013). As expected, knocking down *ChAT* to reduce acetylcholine levels in the MB decreased social space in both sexes, even if more in females (Fig. 4a). Although beyond the scope of this current work, assessing the role of sNPF in social spacing should be pursued; as its role so far has mostly been associated with food search, metabolism and circadian rhythm (for review, as well as some aspects of male behavior (Kim et al. 2013).

Dopamine has been shown to regulate social spacing (Fernandez et al. 2017; Xie et al. 2018; Yost et al. 2024), and the MB lobes are modulated primarily by dopamine (Aso et al. 2014; Li et al. 2020). We also found that manipulating the expression of all dopamine receptor types in the MB increased male social space, without a strong effect in females, if any (Fig. 5a–d). Only *Dop1R2* and *DopEcR* knockdowns also increased female social space, with a very small effect size (Fig. 5b and d), which was at the upper end of how close flies can group together, suggesting a ceiling effect.

Overall, our results indicate that MB acetylcholine release influences social space in both sexes, although more in females, but dopaminergic inputs into the MB differentially modify female and male social space preference. It was interesting to observe that all 4 types of Drosophila dopamine receptors (Dop1R1, Dop1R2, Dop2R, and DopEcR) had the same effect on social space while each receptor plays unique roles in different fly behaviors, such as sleep, courtship, feeding, and memory (Karam et al. 2019).

There is also some modulation of the MB from GABAergic anterior paired lateral neurons (Zhou et al. 2019) and serotonergic dorsal paired medial neurons (Lee et al. 2021). Future work will aim to identify which MB neuromodulators (neuropeptides, serotonin, GABA), MB lobe compartments, dopaminergic neurons (DANs), and mushroom body output neurons (MBONs) are important in social space determination. Indeed, DANs innervate specific compartments within the MB lobes to modulate synaptic transmission between intrinsic MB Kenyon cells and MBONs (Aso et al. 2014). Approximately 130 DANs target the MB, and 20 of the 22 distinct cell types (Aso et al. 2014) arise from the PAM and PPL1 clusters, while the 2 other types originating from the PPL2 cluster and project to the MB calyx. In addition, Dop1R1 and Dop2R are broadly distributed across Kenyon cell membranes, DopEcR is enriched near the calyx, whereas Dop1R2 localizes to the peduncle and distal axonal regions (Kondo et al. 2020). As all DopRs are involved in social spacing in males, we propose a hypothesis generating model (Fig. 8), in which PPL1 and PAM DANs are relaying signals of importance in social spacing. However, as both Nlg3 and DopEcR are located in the calyces of the mushroom bodies, the PPL2ab cluster might also be very important for social spacing.

### Involvement of *nlg3* and fruP1 neurons in social space determination

Beyond the results we report here, many genetic and neural manipulations have sex-specific effects on Drosophila social space, despite both sexes responding to changes in their social environment in similar ways. For example, age, mating status, and isolation all lead to increased social space in both sexes (Simon et al. 2012; Brenman-Suttner et al. 2018; Yost et al. 2024). We thus examined the involvement of sexually dimorphic *fruP1* neurons in social space. Again, we saw sex differences in response to modulation of *fruP1* neuronal transmission. In males, hyperactivating *fruP1* neurons caused flies to settle further apart, but when silencing these neurons, males were closer together (Fig. 6). Females also settled further apart when *fruP1* neurons were hyperactivated, but their social space did not change when these neurons were silenced (Fig. 6). This indicates that increasing and decreasing neuronal activity of *fruP1* neurons in males can change in their social space, but for females, only an increase in *fruP1* neuron activity modifies their social space. The basal transmission of these neurons affecting female social space might be very low, and therefore cannot be silenced further; only hyperactivation changes this resting state and leads to females settling further in a group setting.

After observing the importance of *fruP1* neurons in Drosophila social space, we then examined the molecular players within these neurons that may contribute to these behavioral changes. Acetylcholine and GABA are reportedly produced in *fru* neurons (Palmateer et al. 2023). We show that release of either GABA or acetylcholine from *fruP1* neurons is important for proper social spacing in both sexes. Knocking down *nlg3* in *fruP1* neurons had a sexually dimorphic effect, with no effect on males but females settling closer (Fig. 7a). As mentioned earlier, *fruP1* neurons affecting social space are cholinergic, in both males and females. The gene *fruP1* is expressed in the mushroom body (Yu et al. 2010; von Philipsborn et al. 2014), specifically, it is enriched in gamma lobes (Shih et al. 2019; Palmateer et al. 2023), but also detected in alpha-beta lobes (Palmateer et al. 2023); an expression pattern established early in development (Ito et al. 1996). Interestingly, according to the Fly Atlas database (Li et al. 2022), some MB neurons simultaneously express *fruP1* and *nlg3*. Based on this and our own data, we propose that it could be the Kenyon cell subset of *nlg3* neurons also expressing *fruP1* that are involved in social space determination (hypothesis generating model Fig. 8).

By dissecting the molecular players within these neuronal subsets, we identify some of the inputs and outputs of the MB that influence fly social spacing. Although we manipulate neurotransmission in adults to show that specific brain structures are involved in social spacing, we cannot exclude that the effects of *nlg3* in particular, and of the different neurotransmitters, could indicate a role associated with development of those brain structures and not their functionality, as we tested using RNAi tools driven in a tissue specific manner, but without temporal control.

Nevertheless, using this initial set of behavioral data, we propose a hypothesis generating model of the neural substrates regulating social space, highlighting where sexual dimorphic integration of cues might be taking place (Fig. 8). In addition, the data reported in this study allowed us to propose a more complex putative circuitry (reported in Robinson et al. 2025), using additional information gathered from FlyWire Codex (Zheng et al. 2018; Buhmann et al. 2021; Dorkenwald et al. 2024; Eckstein et al. 2024; Schlegel et al. 2024; Lin et al. 2024; Matsliah et al. 2024).

The conserved role of neuroligins in synaptic organization across species highlights Drosophila as a valuable model for dissecting fundamental mechanisms underlying social behavior.

This first description of the neural substrates regulating social space serves as a basis for completing the underlying circuitry. We next need to confirm the developmental stage at which the impairments on social spacing we report are taking place. In addition, now that we have a broad picture of the neural substrates involved, we need more specificity: for example, we observed that dopaminergic signaling into the MB influences social space but still need to identify which dopaminergic clusters are playing a role (Li et al. 2020). We can similarly test the known dopaminergic subsets that innervate the MB (PPL1, PAM, and PPL2ab). We also need to identify which MB input(s) play a role in female social space determination. Finally, we show the role of acetylcholine signaling from the MB as a social space modulator in both sexes (unsurprisingly), but we have yet to identify which mushroom body output neurons (MBONs), and which specific MB compartments are associated with these behavioral changes (Fig. 8). To establish downstream circuits that may influence this social behavior, we would have to assess which of the known ∼35 MBONs (Li et al. 2020; Scaplen et al. 2021) play a role in social space determination.

These findings may provide insight into how altered synaptic organization and sexually dimorphic neural processing contribute to variability in social behaviors associated with neurodevelopmental disorders.

In conclusion, our findings identify a sexually dimorphic neural circuitry underlying Drosophila social spacing that involves Neuroligin 3, the mushroom bodies, protocerebral bridge, optic lobes, and fruitless-expressing neurons. Although males and females display similar behavioral outcomes following some manipulations, the underlying neural substrates and neurotransmitter systems differ between sexes. Our results further demonstrate that neuroligin-dependent synaptic signaling contributes to the integration of social cues and modulation of group behavior. Given the conserved roles of neuroligins in synapse organization and the association of human NLGN genes with autism spectrum disorders, this work provides a foundation for understanding how sex-specific neural circuits and synaptic mechanisms regulate social behavior across species.

## Supplementary Material

iyag243_Supplementary_Data

## Data Availability

The datasets analyzed for this study are deposited in the Dryad repository (DOI: 10.5061/dryad.931zcrk01). Supplemental material available at GENETICS online.
