# Supplementary material for: Neuroligin 3 highlights the sexually dimorphic neural substrates of Drosophila social spacing
Source: Genetics. 2026 Sep 8;234(3):iyag243. doi: 10.1093/genetics/iyag243 (PMC13732740; doi:10.1093/genetics/iyag243)
Supplement: Supplementary file 1 [file iyag243_supplementary_data.pdf]

Supplemental material for:

Neuroligin 3 highlights the sexually dimorphic neural substrates of Drosophila social spacing

Authors:

Robinson JW[1], Bechard AT[1], Evans MR[1], Kurbaj J[1†], Ataei R[1], Mosuro K[1†], Pillay S[1†], Lin DS[1†], Sahota A[1†], de Belle JN[1†], Robinson GI[2], Yost, RT[1,3], Moehring AJ[1], Simon, AF[1*]

Affiliations:

1. Department of Biology, University of Western Ontario,
   1151 Richmond Street
   London, Ontario, Canada N6A 5B7

2. Department of Biological Sciences, University of Lethbridge,
   4401 University Drive
   Lethbridge, Alberta, T1K 3M4

3. London Health Sciences Centre Research Institute,
   800 Commissioners Rd E
   London, ON
   N6A 5W9

† Undergraduate students at time of data gathering
* Correspondence: asimon28@uwo.ca (AFS)

Running head

Drosophila social spacing neural substrates

Keywords:
Drosophila, social space, social behaviour, neural substrates, *neuroligin*, mushroom bodies, *fruitless*, acetylcholine, GABA, dopamine, and dopamine receptors

Supplemental Results

Neuroligin 3 protein levels after nlg3-RNAi knockdown

We aimed to confirm whether the RNAi line we created resulted in reduced Nlg3 protein level. We were able to use what was left from our anti-Nlg3 antibody to perform only one Western blot experiment, on mixed sex flies, prior to outcrossing the RNAi lines to Canton-S. We used an Elav-Gal 4 driver to direct *nlg3*-RNAi pan-neuronally because at that time we had not yet acquired the *nlg3*-Gal4 driver. As previously reported (XIE *et al.* 2023; WU *et al.* 2018), there are at least four protein isoforms for Nlg3, and they show on a western blot as two bands of similar weight, quantified together as the full-length isoform (FL) and two bands of lesser molecular weight, quantified together as the short isoform (S). In **Supp. Fig. 3B**, a reduction in protein levels of around 50% is observed when comparing the UAS-*nlg3*-RNAi /+ line to *nlg3*-Gal4 > *nlg3* RNAi line. In this background, the 2 protein isoforms are found in similar amounts, contrary to what we observed in a Canton-S background, where the S forms are fewer than the FL forms (YOST *et al.* 2020).

Neuroligin 3 expression in the optic lobes and the protocerebral bridge modulates fly climbing performance

We used a climbing assay to assess fly locomotion and their ability to perform a climbing startle response; this is done to ensure that all flies could perform in the social space assay. Any fly line with individuals able to reach the top vial (performance index, PI > 0) in 15 seconds does not have impairments that prevent testing in the social space assays (MCNEIL *et al*. 2015; YOST *et al.* 2020).

While *nlg3* knockdown in the MB did not affect climbing, (**Supp. Fig. 2A**), there was a reduction in climbing when *nlg3* was knocked down in each of the PB and OL (**Supp. Fig. 2B,C**). However, flies from each line were able to climb and therefore were able to perform the social space assay.

Hyperactivation and silencing nlg3-expressing neurons, independently or simultaneously, changes fly social space in a sex-specific manner

There was no reduction in climbing when hyperactivating or silencing the MB or PB neurons (**Supp. Fig. 3A,B**), but we did observe a slight reduction in climbing when hyperactivating *nlg3*-expressing neurons in males only (**Supp. Fig. 3C**).

Neurons expressing the fruitless gene under the P1 promotor are involved in sexually dimorphic responses in fly social spacing

We used the *fruP1-Gal4* driver to manipulate neuron transmission. Importantly, we saw a reduction in climbing in both males and females when hyperactivating (Two-way ANOVA – Effect of transmission: $F_{1,32} = 153.6$, $p < 0.0001$) and silencing (Two-way ANOVA – Effect of transmission: $F_{1,32} = 33.54$, $p = 0.0102$) *fruP1* neurons (**Supp. Fig. 5**).

To assess the role of Nlg3 in *fruitless*-expressing neurons, we drove an RNAi against *nlg3* with the *fruP1* driver. We observed a reduction in male climbing when knocking down *nlg3* in *fruP1* neurons (**Supp. Fig. 6A**; Two-way ANOVA – Effect of genotype: $F_{1,42} = 12.92$, $p = 0.0008$). Next, we investigated neurotransmitters in *fruP1* neurons that may be involved in climbing. Knocking down *TH*, the rate limiting-enzyme in the pathway of dopamine synthesis, in *fruP1* neurons decreased climbing in both males and females; this indicates the importance of

dopamine released from *fruP1* neurons in fly climbing (**Supp. Fig. 6B**; Two-way ANOVA – Effect of genotype: $F_{1,31} = 36.21$, $p < 0.0001$). Reducing acetylcholine levels by knocking down *ChAT* in *fruP1* neurons also decreases fly climbing (**Supp. Fig. 6C;** Two-way ANOVA – Effect of genotype: $F_{1,25} = 26.26$, $p < 0.0001$), but not reducing GABA by knocking down *GAD1* in *fruP1* neurons (**Supp. Fig. 6D**; Two-way ANOVA – Effect of genotype: $F_{1,38} = 2.111$, $p = 0.1544$).

Supplementary discussion

As previously reported (JIANG *et al.* 2020; YOST *et al.* 2024), we did not observe any correlation in our experiments between variations in climbing performance index and changes in social space. We have tested climbing in our studies of social spacing (MCNEIL *et al*. 2015; YOST *et al.* 2020; this work and unpublished work) because we want to ensure that major locomotor deficits do not interfere with our social spacing assay. In less successful situations (flies not able to climb and with major locomotion problems), we had to adapt the set-up of the assay, to test the flies horizontally, as in CASTELL-NOBAU *et al.* (2019).

However, in the condition of our test, we report observing no correlation in multiple studies. The fact that we and others report no correlation between deficits in locomotion and preferred social space is unexpected, and fascinating. JIANG *et al.* (2020) report in their detailed description of fly cluster formation that locomotion deficits do not underlie deficits in social clustering. We show here that decreased climbing does not underlie successful social spacing.

Using our data, we statistically confirm that climbing assay results do not predict social spacing preferences (**Supp. Table 5&6**).

The climbing assay is performed under light, which can explain why disrupting the OL (function or development) could also alter the flies phototaxic behaviour, and thus their climbing abilities, as seen when *nlg-3* is down regulated in that structure.

The role of the PB in locomotion is well established (REF), and we do see climbing altered when hyperactivating that structure. It is more surprising to observe that hyperactivating *nlg3*-expressing cells, including those in the PB, appears to only affect males, and not females; this underscores another sexually dimorphic effect of manipulating *nlg3* expressing cells.

The MB is not known to be a primary locomotor centre, which may explain why knocking down *nlg3* and manipulating neuronal transmission in this region had no effect on climbing behaviour. The only times MB-targeted manipulations influenced climbing was when RNAi lines for Dop1R1, Dop2R, and DopEcR were used. Altering Dop1R1 expression in the MB led to increased climbing, which aligns with the known role of this receptor in modulating locomotion; indeed reducing Dop1R1 expression has been shown to increase larval movement (KARAM *et al.* 2019; SILVA *et al.* 2020), and when combined with the results presented here, this suggests that Dop1R1 may play an inhibitory role in locomotor responses. Dop2R and DopEcR have not been directly shown to influence locomotion, but manipulations to these receptors may cause corresponding changes in the expression levels of Dop1R1, which could account for the increased climbing observed.

The *fru* gene is expressed in motor neurons (RIDEOUT *et al.* 2007), and the male-specific muscle "muscle of Lawrence" (MOL), as well as in many neuronal and non-neuronal cells (AUER and BENTON 2016). So, unsurprisingly, in addition to abnormal courtship, locomotion defects have been reported for males $fru^{\Delta P1}$ (NEVILLE *et al.* 2021), and with an RNAi against *fru*, driven in the oenocytes (SUN *et al.* 2023). In our hands, acetylcholine release alters climbing in *fruP1* neurons in both sexes, and such a release is involved in Tachykinin/Substance P release from *fru* neurons, in males, in aggression behaviour (ASAHINA *et al.* 2014).

Supplemental Methods

Supplemental fly line

The pan-neuronal Elav-Gal4 driver w+; elav-Gal4/CyO was obtained from Bloomington Drosophila Stock Center, BL #8765, and outcrossed in Canton-s background 5 times.

Microscopy

Fluorescent imaging was performed on an Imager Z1 Zeiss compound fluorescent microscope and confocal imaging was performed on a Zeiss LSM 5 Duo Vario confocal microscope in the Biotron Imaging Facility (Western University). Images within individual fluorescent figures were captured and processed with identical exposure time and magnification.

Western Blot and Protein Analysis

Performed as described in YOST *et al.* (2020). In short, 20 mixed male and female heads, were homogenized in 1X Laemmli sample buffer, with 1% dithiothreitol (BioRad, Mississauga, ON, Canada). Protein lysates were separated on a 10% SDS-polyacrylamide gel (TGX FastCast Stain-Free gels; BioRad, Mississauga, ON, Canada) and electro-transferred to a nitrocellulose membrane. Proteins were incubated with a polyclonal guinea pig anti-Nlg3 antibody (1:4000) overnight at 4◦C, followed by horseradish peroxidase conjugated secondary antibodies (BioRad, Mississauga, ON, Canada; 1:10,000) and visualized using the ClarityMax Western enhanced chemiluminescence substrate (BioRad, Mississauga, ON, Canada). Western blots were analyzed with ImageLab software (BioRad, Mississauga, ON, Canada). All treatments were normalized to total protein by visualizing proteins on the membrane directly, following UV illumination of the blots before enhanced chemiluminescence addition and detection occurred. All bands below the autofluorescence (likely resulting from the eye pigments) were included in the normalization.

Supplemental References

Asahina, K., K. Watanabe, B. J. Duistermars, E. Hoopfer, C. R. Gonzalez *et al.*, 2014 Tachykinin-expressing neurons control male-specific aggressive arousal in Drosophila. Cell 156**:** 221-235.

Auer, T. O., and R. Benton, 2016 Sexual circuitry in Drosophila. Curr Opin Neurobiol 38**:** 18-26.

Castells-Nobau, A., I. Eidhof, M. Fenckova, D. B. Brenman-Suttner, J. M. Scheffer-de Gooyert et al., 2019 Conserved regulation of neurodevelopmental processes and behavior by FoxP in Drosophila. PLoS One 14: e0211652.

Jiang, L., Y. Cheng, S. Gao, Y. Zhong, C. Ma *et al.*, 2020 Emergence of social cluster by collective pairwise encounters in Drosophila. eLife 9.

Karam, C. S., S. K. Jones and J. A. Javitch, 2019 Come Fly with Me: An overview of dopamine receptors in *Drosophila melanogaster*. Basic & Clinical Pharmacology & Toxicology.

McNeil, A., S. N. Jolley, A. A. Akinleye, M. Nurilov, Z. Rouzyi *et al.*, 2015 Conditions affecting social space in *Drosophila melanogaster*. J. Vis. Exp. 105**:** e53242.

Neville, M. C., A. Eastwood, A. M. Allen, A. de Haan, T. Nojima *et al.*, 2021 Generation and characterization of f*ruitless P1* promoter mutant in *Drosophila melanogaster*. J Neurogenet 35**:** 285-294.

Rideout, E. J., J. C. Billeter and S. F. Goodwin, 2007 The sex-determination genes fruitless and doublesex specify a neural substrate required for courtship song. Curr Biol 17**:** 1473-1478.

Silva, B., S. Hidalgo and J. M. Campusano, 2020 Dop1R1, a type 1 dopaminergic receptor expressed in Mushroom Bodies, modulates Drosophila larval locomotion. PLoS One 15: e0229671.

Wu, J., N. Tao, Y. Tian, G. Xing, H. Lv *et al.*, 2018 Proteolytic maturation of Drosophila Neuroligin 3 by tumor necrosis factor α-converting enzyme in the nervous system. Biochimica et Biophysica Acta (BBA) - General Subjects.

Xie, H., S. Liu, Y. Fu, Q. Cheng, P. Wang *et al.*, 2023 Nuclear access of DNlg3 c-terminal fragment and its function in regulating innate immune response genes. Biochem Biophys Res Commun 641**:** 93-101.

Yost, R. T., J. W. Robinson, C. M. Baxter, A. M. Scott, L. P. Brown *et al.*, 2020 Abnormal social interactions in a Drosophila mutant of an autism candidate gene: *neuroligin 3*. Int J Mol Sci 21.

Supplemental Tables

Fly Line Name	Identifier	Source	Genotype	Description
Control Strain				
Cs	64346	Seymour Benzer	+/+	Laboratory strain
attP2	36303	BDSC	*y[1] v[1]; P{y[+t7.7]=CaryP}attP2*	*attP* in the text.<br>Background stock used as control for DopR TRiP RNAi lines
nlg3 insertions (Outcrossed 5X in Cs)				
MiMIC-nlg3	58509	BDSC	*y[1]w[*];Mi{y[+mDint2]=MIC}Nlg3[MI08924]*	Insertion in 1st intron, contains *EGFP*
nlg3-Gal4	76134	BDSC	*y[1] w[*]; Mi{Trojan-GAL4.2}Nlg3[MI00445-TG4.2]*	Insertion in 3rd intron
Lines used for the generation of the *UAS-nlg 3 RNAi*				
	25709	BDSC	*y[1] v[1] P{y[+t7.7]=nos- phiC31\int.NLS}X; P{y[+t7.7]=CaryP}attP40*	Injection strain
	3707	BDSC	*w[1118]/Dp(1;Y)y[+]; CyO/nub[1] b[1] sna[Sco] lt[1] stw[3]; MKRS/TM6B, Tb[1]*	Balancer line
Gal4 Drivers (Outcrossed 5X in Cs)				
MB-Gal4	49265<br>R15E01	BDSC<br>FlyLight	*w[1118]; P{y[+t7.7] w[+mC]=GMR15E01-GAL4}attP2*	Mushroom body expression under the enhancer of *rut*
PB-Gal4	50422<br>R55G08	BDSC<br>FlyLight	*w[1118]; P{y[+t7.7] w[+mC]=GMR55G08-GAL4}attP2*	Protocerebral bridge expression under the enhancer of *cmpy*
OL-Gal4	49234<br>*R10B01*	BDSC<br>FlyLight	*w[1118]; P{y[+t7.7] w[+mC]=GMR10B01-GAL4}attP2*	Optic lobe expression under the enhancer of *crp*
fruP1-Gal4	66696	BDSC	*TI{GAL4}fru[GAL4.P1.D]/TM3, Sb[1]*	Gene trap: expresses Gal4 under control of *fruP1*
UAS Effectors				
mCD8-GFP	6314	BDSC	*y[1] w[*] P{w[+mC] =UAS-mCD8::GFP.L} Ptp4E[LL4]P{w[+mW.hs]=GawB}lz[gal4]*	Expresses Membrane-bound GFP
UAS-TrpA1	26263	BDSC	*w[*]; P{y[+t7.7] w[+mC]=UAS-TrpA1(B).K}attP16*	Temperature control of neuron activation
$shit^{ts}$	44222	BDSC	*w[*]; P{w[+mC]=UAS-shi[ts1].K}3*	Temperature control of neuron silencing
nlg3-RNAi	n/a	This paper	w^{1118}*; P{v[+]=UAS-nlg3}*	RNAi knockdown of *nlg3*, outcrossed 5X in Cs background
DopEcR-RNAi	31981	BDSC	*y[1] v[1]; P{y[+t7.7] v[+t1.8]=TRiP.JF03415}attP2*	
Dop1R1-RNAi	31765	BDSC	*y[1] v[1]; P{y[+t7.7] v[+t1.8]=TRiP.HM04077}attP2*	RNAi knockdown of dopamine receptors
Dop1R2-RNAi	26018	BDSC	*y[1] v[1]; P{y[+t7.7] v[+t1.8]=TRiP.JF02043}attP2*	
Dop2R-RNAi	26001	BDSC	*y[1] v[1]; P{y[+t7.7] v[+t1.8]=TRiP.JF02025}attP2*	
ChAT25-RNAi	25856	BDSC	*y[1] v[1]; P{y[+t7.7] v[+t1.8]=TRiP.JF01877}attP2*	RNAi knockdown of *choline acetyltransferase*
Gad51-RNAi	51794	BDSC	*y[1] v[1]; P{y[+t7.7] v[+t1.8]=TRiP.HMC03350}attP40*	RNAi knockdown of *glutamate decarboxylase 1*
TH-RNAi	n/a	Mark Wu (Johns Hopkins)	*w[*];;UAS-TH RNAi 3#G / TM6, Sb*	RNAi knockdown *of tyrosine hydroxylase*

Supplemental Table 1: Genotype table of fly lines used. BDSC: Bloomington Drosophila Stock Center

Experiment	Figure	Effect	Df	F	p<br>*corrected p**	Šídák's post hoc		
Social space						**Comparison Group**	**Compared with**	***p***
MB>nlg3-RNAi	2A	**Sex**	(1,39)	**13.97**	**0.0006**	*Male*		
					0.0012*	MB>RNAi	RNAi/+	0.3492
		**Genotype**		**14.71**	**0.0004**	*Female*		
					0.0008*	**MB>RNAi**	**RNAi/+**	**0.0005**
		Sex * Genotype		4.009	0.0523			0.001*
PB>nlg3-RNAi	2B	**Sex**	(1,40)	**14.90**	**0.0004**	*Male*		
					0.0008*	PB>RNAi	RNAi/+	0.2497
		**Genotype**		**19.02**	**<0.0001**	*Female*		
					<0.0002*	**PB>RNAi**	**RNAi/+**	**0.0001**
		**Sex * Genotype**		**5.607**	**0.0228**			0.0002*
					0.0456*			
OL>nlg3-RNAi	2C	**Sex**	(1,31)	**31.29**	**<0.0001**	*Male*		
		**Genotype**		**7.191**	**0.0116**	OL>RNAi	RNAi/+	0.4048
		Sex * Genotype		0.9424	0.3392	*Female*		
						**OL>RNAi**	**RNAi/+**	**0.0320**
MB>TrpA1	3A	**Sex**	(1,47)	**5.382**	**0.0247**	*Male MB>Trp*		
		**Neural Transmission**		**7.395**	**0.0091**	Activated	Inactivated	0.2437
		Sex * Neural Transmission		0.172	0.6796	*Female MB>Trp*		
						**Activated**	**Inactivated**	**0.0442**
MB>Shi^{ts}	3A	Sex	(1,30)	0.680	0.4161	*Male MB>Shi*		
		**Neural Transmission**		**14.40**	**0.0007**	**Activated**	**Inactivated**	**0.0105**
		Sex * Neural Transmission		0.344	0.5617	*Female MB>Shi*		
						Activated	Inactivated	0.0518
PB>TrpA1	3B	Sex	(1,74)	39.94	0.0868	*Male PB>Trp*		
		Neural Transmission		17.72	0.2018	Activated	Inactivated	0.9999
		Sex * Neural Transmission		13.62	0.1953	*Female PB>Trp*		
						Activated	Inactivated	0.1382
PB>Shi^{ts}	3B	Sex	(1,31)	0.6335	0.4321	*Male PB>Shi*		
		Neural Transmission		3.443	0.0730	Activated	Inactivated	0.8897
		Sex * Neural Transmission		1.483	0.2325	*Female PB>Shi*		
						Activated	Inactivated	0.0596
nlg3-Gal4>TrpA1	3C	Sex	(1,28)	2.380	0.1341	*Male Trojan>Trp*		
		**Neural Transmission**		**12.70**	**0.0013**	Activated	Inactivated	0.2214
		Sex * Neural Transmission		1.366	0.2523	*Female Trojan>Trp*		
						**Activated**	**Inactivated**	**0.0029**
nlg3-Gal4>Shi^{ts}	3C	**Sex**	(1,22)	**4.666**	**0.0419**	*Male MB>Trp*		
		**Neural Transmission**		**5.998**	**0.0228**	Activated	Inactivated	0.9996
		**Sex * Neural Transmission**		**6.169**	**0.0211**	*Female MB>Trp*		
						**Activated**	**Inactivated**	**0.0042**
MB>CHAT25-RNAi	4	**Sex**	(1,32)	**41.09**	**<0.0001**	*Male*		
		**Genotype**		**11.19**	**0.0021**	MB>RNAi	RNAi/+	0.4588
		Sex * Genotype		3.020	0.0919	*Female*		
						**MB>RNAi**	**RNAi/+**	**0.0022**
MB>Dop1R1-RNAi	5A	**Sex**	(1,32)	**201.7**	**<0.0001**	*Male*		
					<0.0002*	**MB>RNAi**	**MB/+**	**<0.0001**
		**Genotype**		**68.23**	**<0.0001**			<0.0002*
					<0.0002*	*Female*		
		**Sex * Genotype**		**24.48**	**<0.0001**	MB>RNAi	MB/+	0.0505
					<0.0002*			0.101*
MB>Dop1R2-RNAi	5B	**Sex**	(1,32)	**139.4**	**<0.0001**	*Male*		
					<0.0002*	MB>RNAi	**MB/+**	**0.0225**
		**Genotype**		**7.401**	**0.0105**			0.045*
					0.021*	*Female*		
		Sex * Genotype		1.168	0.288	MB>RNAi	MB/+	0.4447
MB>Dop2R-RNAi	5C	**Sex**	(1,32)	**120.7**	**<0.0001**	*Male*		
					<0.0002*	**MB>RNAi**	MB/**+**	**<0.0001**
		**Genotype**		**41.1**	**<0.0001**			<0.0002*
					<0.0002*	*Female*		
		**Sex * Genotype**		**7.448**	**0.0102**	**MB>RNAi**	MB/**+**	0.3223
					0.0204*			
MB>DopEcR-RNAi	5D	**Sex**	(1,32)	**139.3**	**<0.0001**	*Male*		
					<0.0002*	**MB>RNAi**	**MB/+**	**0.0103**
		**Genotype**		**8.905**	**0.0054**			0.101*
					0.0108*	*Female*		
		Sex * Genotype		1.588	0.2167	MB>RNAi	MB/+	0.4098
fruP1>TrpA1	6A	**Sex**	(1,33)	**5.168**	**0.0296**	*Male fruP1>Trp*		
		**Neural Transmission**		**22.65**	**<0.0001**	**Activated**	**Inactivated**	**0.0241**
		Sex * Neural Transmission		0.6987	0.4092	*Female fruP1>Trp*		
						**Activated**	**Inactivated**	**0.0004**
fruP1>Shi^{ts}	6B	Sex	(1,30)	0.5671	0.4573	*Male fruP1>Trp*		
		Neural Transmission		2.350	0.1358	**Activated**	**Inactivated**	**0.0453**
		Sex * Neural Transmission		3.451	0.0730	*Female fruP1>Trp*		
						Activated	Inactivated	0.9676
fruP1>nlg3-RNAi	7A	**Sex**	(1,57)	**15.57**	**0.0002**	*Male*		
		Genotype		**16.27**	**0.0002**	*fruP1*>RNAi	RNAi/+	0.3363
		**Sex * Genotype**		**4.09**	**0.0478**	***Female***		
						***fruP1*>RNAi**	**RNAi/+**	**<0.0001**
fruP1>TH-RNAi	7B	**Sex**	(1,29)	**32.36**	**<0.0001**	*Male*		
		Genotype		0.413	0.5255	*fruP1*>RNAi	*fruP1*/+	0.9938
		Sex * Genotype		0.6034	0.4436	*Female*		
						fruP1>RNAi	*fruP1*/+	0.5709
fruP1>CHAT25-RNAi	7C	**Sex**	(1,47)	**16.12**	**0.0002**	***Male***		
		**Genotype**		**99.11**	**<0.0001**	***fruP1*>RNAi**	**RNAi/+**	**<0.0001**
		Sex * Genotype		0.7134	0.4026	***Female***		
						***fruP1*>RNAi**	**RNAi/+**	**<0.0001**
fruP1>GAD51-RNAi	7D	**Sex**	(1,42)	**11.81**	**0.0013**	***Male***		
		**Genotype**		**8.478**	**0.0057**	***fruP1*>RNAi**	**RNAi/+**	**0.0363**
		Sex * Genotype		0.2264	0.6367	*Female*		
						fruP1>RNAi	RNAi/+	0.1897

Supplemental Table 2. All Two-way ANOVAs performed for social space. All p-values with a result less than 0.05 are bolded.

*when same controls were used for more than 1 experiments, and p <0.05, we added a p corrected for multiple comparisons

Experiment	Supp. Figure	Effect	*Df*	*F*	*P* corrected *p**	***Šídák's post hoc***		
Climbing						Comparison Group	Compared with	*p*
TrpA1/+	6D	Sex	(1,20)	2.169	0.156	*Male*		
		**Temperature**		**8.342**	**0.0091**	25°C	29°C	0.1627
		Sex * temperature		0.106	0.7484	*Female*		
						25°C	29°C	0.0674
Shi^{ts}/+	6E	Sex	(1,20)	0.5381	0.4717	*Male*		
		Temperature		0.7418	0.3993	25°C	29°C	0.9089
		Sex * temperature		0.0929	0.7637	*Female*		
						25°C	29°C	0.6629
+/+	6F	Sex	(1,20)	0.224	0.6413	*Male*		
		Temperature		0.895	0.3553	25°C	29°C	0.9972
		Sex * temperature		1.083	0.3104	*Female*		
						25°C	29°C	0.3200
MB>nlg3-RNAi	7A	Sex	(1,26)	0.1363	0.7150	*Male*		
		Genotype		2.146	0.1549	MB>RNAi	RNAi/+	0.1714
		Sex * Genotype		0.8841	0.3557	*Female*		
						MB>RNAi	RNAi/+	0.9231
PB>nlg3-RNAi	7B	Sex	(1,23)	1.349	0.2573	*Male*		
		**Genotype**		**52.07**	**<0.0001**	**PB>RNAi**	**RNAi/+**	**<0.0001**
					<0.0001*			<0.0001*
		**Sex * Genotype**		**4.505**	**0.0448**	*Female*		
					0.0896*	**PB>RNAi**	**RNAi/+**	**0.0034**
								0.0068*
OL>nlg3-RNAi	7C	Sex	(1,16)	3.924	0.0651	*Male*		
		**Genotype**		**88.04**	**<0.0001**	**OL>RNAi**	**RNAi/+**	**<0.0001**
		**Sex * Genotype**		**10.45**	**0.0052**	*Female*		
						**OL>RNAi**	**RNAi/+**	**0.0010**
MB>TrpA1	8A	Sex	(1,18)	2.591	0.1249	*Male MB>Trp*		
		Neural Transmission		4.359	0.0513	Activated	Inactivated	0.4658
		Sex * Neural Transmission		0.1717	0.6835	*Female MB>Trp*		
						Activated	Inactivated	0.1574
MB>Shi^{ts}	8A	**Sex**	**(1,19)**	**18.95**	**0.0003**	*Male MB>Shi*		
		Neural Transmission		0.0243	0.8776	Activated	Inactivated	0.9857
		Sex * Neural Transmission		0.0029	0.9575	*Female MB>Shi*		
						Activated	Inactivated	0.9969
PB>TrpA1	8B	Sex	(1,12)	0.5633	0.4674	*Male PB>Trp*		
		Neural Transmission		0.3293	0.5767	Activated	Inactivated	0.7708
		Sex * Neural Transmission		0.1301	0.7246	*Female PB>Trp*		
						Activated	Inactivated	0.9862
PB>Shi^{ts}	8B	Sex	(1,15)	3.534	0.0797	*Male PB>Shi*		
		Neural Transmission		0.3622	0.5563	Activated	Inactivated	0.8414
		Sex * Neural Transmission		0.0168	0.8985	*Female PB>Shi*		
						Activated	Inactivated	0.9375
nlg3-Gal4>TrpA1	8C	Sex	(1,20)	0.5228	0.4780	*Male Trojan>Trp*		
		**Neural Transmission**		**4.775**	**0.0410**	Activated	Inactivated	0.0750
		Sex * Neural Transmission		1.173	0.2917	*Female Trojan>Trp*		
						Activated	Inactivated	0.6693
nlg3-Gal4>Shi^{ts}	8C	Sex	(1,19)	0.1124	0.7411	*Male MB>Trp*		
		Neural Transmission		0.2331	0.6348	Activated	Inactivated	0.9245
		Sex * Neural Transmission		0.0011	0.9736	*Female MB>Trp*		
						Activated	Inactivated	0.9367
fruP1>TrpA1	9A	**Sex**	**(1,32)**	**25.10**	**<0.0001**	*Male fruP1>Trp*		
		**Neural Transmission**		**153.6**	**<0.0001**	**Activated**	**Inactivated**	**<0.0001**
		Sex * Neural Transmission		3.408	0.0742	*Female fruP1>Trp*		
						**Activated**	**Inactivated**	**<0.0001**
fruP1>Shi^{ts}	9B	Sex	(1,32)	0.0873	0.7695	*Male fruP1>Trp*		
		**Neural Transmission**		**33.54**	**<0.0001**	**Activated**	**Inactivated**	**<0.0001**
		**Sex * Neural Transmission**		**4.278**	**0.0468**	*Female fruP1>Trp*		
						**Activated**	**Inactivated**	**0.0257**
fruP1>nlg3-RNAi	10A	**Sex**	**(1,40)**	**3.688**	**0.0140**	*Male*		
		**Genotype**		**9.735**	**0.0033**	***fruP1>RNAi***	**RNAi/+**	**0.0031**
		Sex * Genotype		6.601	0.0620	*Female*		
						fruP1>RNAi	RNAi/+	0.6103
fruP1>TH-RNAi	10B	Sex	(1,31)	0.3099	0.5817	***Male***		
		**Genotype**		**18.07**	**0.0002**	***fruP1>RNAi***	RNAi/+	0.0712
		Sex * Genotype		1.229	0.2762	*Female*		
						fruP1>RNAi	**RNAi/+**	**0.0011**
fruP1>CHAT25-RNAi	10C	Sex	(1,25)	18.82	0.1822	*Male*		
		**Genotype**		**26.26**	**<0.0001**	*fruP1>RNAi*	**RNAi/+**	**0.0078**
		Sex * Genotype		0.2810	0.6007	*Female*		
						fruP1>RNAi	**RNAi/+**	**0.0008**
fruP1>GAD51-RNAi	10D	Sex	(1,38)	3.336	0.0757	*Male*		
		Genotype		2.111	0.1544	*fruP1>RNAi*	RNAi/+	0.5132
		Sex * Genotype		0.0010	0.9742	*Female*		
						fruP1>RNAi	RNAi/+	0.5367

Supplemental Table 3. All Two-way ANOVAs performed for climbing experiments presented in Supplemental Figures. All p-values with a result less than 0.05 are bolded.

*when same controls were used for more than 1 experiments, and p <0.05, we added a p corrected for multiple comparisons

**Experiment**	**Supp. Figure**	**Effect**	***Df***	***F***	***p***	***Šídák's post hoc***		
Social space						**Comparison Group**	**Compared with**	***p***
MB/+	5A	**Sex**	**(1,23)**	**73.16**	**<0.0001**	*Male*		
		Temperature		0.181	0.181	25°C	29°C	0.797
		**Sex * temperature**		**4.927**	**0.036**	***Female***		
						**25°C**	**29°C**	**0.040**
OL/+	5B	**Sex**	**(1,28)**	**240.5**	**<0.0001**	*Male*		
		Temperature		0.260	0.613	25°C	29°C	0.5058
		Sex * temperature		0.984	0.329	*Female*		
						25°C	29°C	0.9302
PB/+	5C	Sex	(1,20)	0.098	0.757	*Male*		
		Temperature		0.833	0.372	25°C	29°C	0.905
		Sex * temperature		0.118	0.734	*Female*		
						25°C	29°C	0.621
fruP1/+	5D	**Sex**	**(1,28)**	**49.56**	**<0.0001**	*Male*		
		Temperature		2.512	0.124	25°C	29°C	0.382
		Sex * temperature		0.024	0.877	*Female*		
						25°C	29°C	0.558
TrpA1/+	6A	Sex	(1,15)	0.622	0.442	*Male*		
		Temperature		0.410	0.531	25°C	29°C	0.454
		Sex * temperature		0.826	0.377	*Female*		
						25°C	29°C	0.995
Shi^{ts}/+	6B	Sex	(1,9)	0.0302	0.855	*Male*		
		Temperature		0.0064	0.937	25°C	29°C	0.972
		Sex * temperature		0.2199	0.650	*Female*		
						25°C	29°C	0.891
+/+	6C	Sex	(1,13)	0.0036	0.9528	*Male*		
		Temperature		<0.001	0.9992	25°C	29°C	0.994
		Sex * temperature		0.0058	0.9403	*Female*		
						25°C	29°C	>0.999
MB> nlg3-RNAi	11A	**Sex**	**(1, 64)**	**15.56**	**0.0002**	*Male*		
		**Genotype**	**(2, 64)**	**21.23**	**<0.0001**	*MB> nlg3-RNAi*	*RNAi/+*	0.3882
		**Sex * Genotype**	**(2, 64)**	**7.823**	**0.0009**		MB/+	0.9996
						Female		
						MB> nlg3-RNAi	***RNAi/+***	**<0.0001**
							MB/+	0.699
PB> nlg3-RNAi	11B	**Sex**	**(1, 62)**	**23.29**	**<0.0001**	*Male*		
		**Genotype**	**(2, 62)**	**17.65**	**<0.0001**	*PB> nlg3-RNAi*	*RNAi/+*	0.3749
		**Sex * Genotype**	**(2, 62)**	**3.452**	**0.0379**		PB/+	0.8837
						Female		
						PB> nlg3-RNAi	***RNAi/+***	**<0.0001**
							PB/+	0.9859
OL> nlg3-RNAi	11C	**Sex**	**(1, 47)**	**32.31**	**<0.0001**	*Male*		
		**Genotype**	**(2, 47)**	**3.716**	**0.0318**	*OL> nlg3-RNAi*	*RNAi/+*	0.6702
		Sex * Genotype	(2, 47)	1.380	0.2616		OL/+	0.1649
						Female		
						OL> nlg3-RNAi	*RNAi/+*	0.0698
							OL/+	0.8122
MB> CHAT-RNAi	12	**Sex**	**(1, 46)**	**40.78**	**<0.0001**	*Male*		
		**Genotype**	**(2, 46)**	**7.446**	**0.0016**	*MB> CHAT-RNAi*	*RNAi/+*	0.635
		**Sex * Genotype**	**(2, 46)**	**5.382**	**0.0079**		MB/+	0.9988
						Female		
						MB> CHAT-RNAi	*RNAi/+*	**0.0013**
							MB/+	**0.0003**
MB> Dop1R1-RNAi	13A	**Sex**	**(1, 48)**	**65.51**	**<0.0001**	***Male***		
		**Genotype**	**(2, 48)**	**19.61**	**<0.0001**	***MB> Dop1R1-RNAi***	***RNAi/+***	**<0.0001**
		**Sex * Genotype**	**(2, 48)**	**27.24**	**<0.0001**		**MB/+**	**<0.0001**
						Female		
						MB> Dop1R1-RNAi	***RNAi/+***	0.1849
							**MB/+**	0.3290
MB> Dop1R2-RNAi	13B	**Sex**	**(1, 48)**	**86.24**	**<0.0001**	***Male***		
		**Genotype**	**(2, 48)**	**5.96**	**0.0049**	***MB> Dop1R2-RNAi***	***RNAi/+***	**<0.0001**
		**Sex * Genotype**	**(2, 48)**	**18.71**	**<0.0001**		**MB/+**	0.0593
						Female		
						MB> Dop1R2-RNAi	***RNAi/+***	0.3174
							**MB/+**	0.7352
MB> Dop2R-RNAi	13C	**Sex**	**(1, 48)**	**52.29**	**<0.0001**	***Male***		
		**Genotype**	**(2, 48)**	**12.66**	**<0.0001**	***MB> Dop2R-RNAi***	***RNAi/+***	**<0.0001**
		**Sex * Genotype**	**(2, 48)**	**13.12**	**<0.0001**		**MB/+**	**<0.0001**
						Female		
						MB> Dop2R-RNAi	***RNAi/+***	0.4001
							**MB/+**	0.1760
MB> DopEcR-RNAi	13D	**Sex**	**(1, 48)**	**130.8**	**<0.0001**	*Male*		
		**Genotype**	**(2, 48)**	**8.181**	**0.0009**	*MB> DopEcR-RNAi*	***RNAi/+***	**<0.0001**
		**Sex * Genotype**	**(2, 48)**	**17.73**	**<0.0001**		**MB/+**	0.0090
						Female		
						MB> DopEcR-RNAi	***RNAi/+***	0.5300
							**MB/+**	0.5829
fruP1> nlg3-RNAi	14A	**Sex**	**(1, 91)**	**9.723**	**0.0024**	*Male*		
		**Genotype**	**(2, 91)**	**9.909**	**0.0001**	*fruP1> nlg3-RNAi*	*RNAi/+*	0.6105
		**Sex * Genotype**	**(2, 91)**	**4.063**	**0.0204**		*fruP1/+*	0.3388
						Female		
						fruP1> nlg3-RNAi	***RNAi/+***	**0.0002**
							fruP1/+	**0.0728**
fruP1> TH-RNAi	14B	**Sex**	**(1, 45)**	**73.38**	**<0.0001**	*Male*		
		Genotype	(2, 45)	1.442	0.2472	*fruP1> TH-RNAi*	*RNAi/+*	>0.999
		Sex * Genotype	(2, 45)	2.182	0.1246		*fruP1/+*	0.9996
						Female		
						fruP1> TH-RNAi	*RNAi/+*	0.8025
							fruP1/+	0.3834
fruP1> CHAT-RNAi	14C	**Sex**	**(1, 75)**	**7.594**	**0.0073**	*Male*		
		**Genotype**	**(2, 75)**	**48.34**	**<0.0001**	*fruP1> CHAT-RNAi*	***RNAi/+***	**<0.0001**
		**Sex * Genotype**	**(2, 75)**	**5.876**	**0.0043**		***fruP1/+***	**0.0236**
						Female		
						fruP1> CHAT-RNAi	***RNAi/+***	**<0.0001**
							fruP1/+	**<0.0001**
fruP1> GAD-RNAi	14D	Sex	(1, 59)	2.117	0.151	*Male*		
		**Genotype**	**(2, 59)**	**6.416**	**0.0030**	*fruP1> GAD-RNAi*	*RNAi/+*	0.0718
		**Sex * Genotype**	**(2, 59)**	**6.258**	**0.0034**		*fruP1/+*	0.999
						Female		
						fruP1> GAD-RNAi	*RNAi/+*	0.3493
							fruP1/+	**0.0003**

Supplemental Table 4. ANOVA table for all Two-Way ANOVAs performed for social spacing experiments presented in Supplemental Figures. All p-values with a result less than 0.05 are bolded. Grey highlights indicate a different genetic background that the other genotypes compared and reveal the importance of controlling for genetic background and excluding inappropriate comparisons (See also **Supplemental Table 1**).

Genetic manipulation	Sex	Social Space Figure #	Climbing*	Social Space*
MB>nlg3-RNAi	M	2a	NC	NC
MB>nlg3-RNAi	F	2a	NC	Increased
PB>nlg3-RNAi	M	2b	Decreased	NC
PB>nlg3-RNAi	F	2b	Decreased	Increased
OL>nlg3-RNAi	M	2c	Decreased	Increased
OL>nlg3-RNAi	F	2c	Decreased	Increased
MB>TrpA1	M	3a	NC	Increased
MB>TrpA1	F	3a	NC	Increased
MB>Shi	M	3a	NC	Increased
MB>Shi	F	3a	NC	Increased
PB>TrpA1	M	3b	NC	NC
PB>TrpA1	F	3b	NC	NC
PB>Shi	M	3b	NC	NC
PB>Shi	F	3b	NC	Increased
OL>TrpA1	M	3c	Decreased	Increased
OL>TrpA1	F	3c	Decreased	Increased
OL>Shi	M	3c	NC	NC
OL>Shi	F	3c	NC	Increased
MB>Dop1R1-RNAi	M	5a	Increased	Increased
MB>Dop1R1-RNAi	F	5a	Increased	NC
MB>Dop1R2-RNAi	M	5b	NC	Increased
MB>Dop1R2-RNAi	F	5b	NC	NC
MB>Dop2R-RNAi	M	5c	Increased	Increased
MB>Dop2R-RNAi	F	5c	Increased	NC
MB>DopEcR-RNAi	M	5d	Increased	Increased
MB>DopEcR-RNAi	F	5d	Increased	NC
fruP1>TrpA1	M	6	Decreased	Increased
fruP1>TrpA1	F	6	Decreased	Increased
fruP1>Shi	M	6	Decreased	Decreased
fruP1>Shi	F	6	Decreased	NC
fruP1>nlg3-RNAi	M	7a	Decreased	NC
fruP1>nlg3-RNAi	F	7a	NC	Decreased
fruP1>TH-RNAi	M	7b	Decreased	NC
fruP1>TH-RNAi	F	7b	Decreased	NC
fruP1>ChAT-RNAi	M	7c	Decreased	Decreased
fruP1>ChAT-RNAi	F	7c	Decreased	Decreased
fruP1>GAD1-RNAi	M	7d	NC	Decreased
fruP1>GAD1-RNAi	F	7d	NC	Decreased

Supplemental Table 5. Comparing changes in social space and climbing performances

**Change in performance/behaviour compared to control: NC no change.*

Supp. Table 6

	No change social space	Increased social space	Decreased social space	***Row Totals***
No change climbing	6 (6.263) [0.0111]	8 (8.053) [0.0003]	3 (2.684) [0.0372]	17
Decreased climbing	5 (5.526) [0.0501]	7 (7.105) [0.016]	3 (2.368) [0.1684]	15
Increased climbing	3 2.211 [0.2820]	3 (2.842) [0.0088]	0 (0.947) [0.9474]	6
Column Totals	14	18	6	**38 (Grand Total)**

Supplemental Table 6: Contingency table with the following information extracted from **Supplemental table 3**: the observed number of experiments, then the following calculations (the expected number of experiments if variable were independent) and [the chi-square statistic for each cell].
Chi-square (df: 4, n= 38)= 1.5068 , p=0.8254.

Supplemental Figures

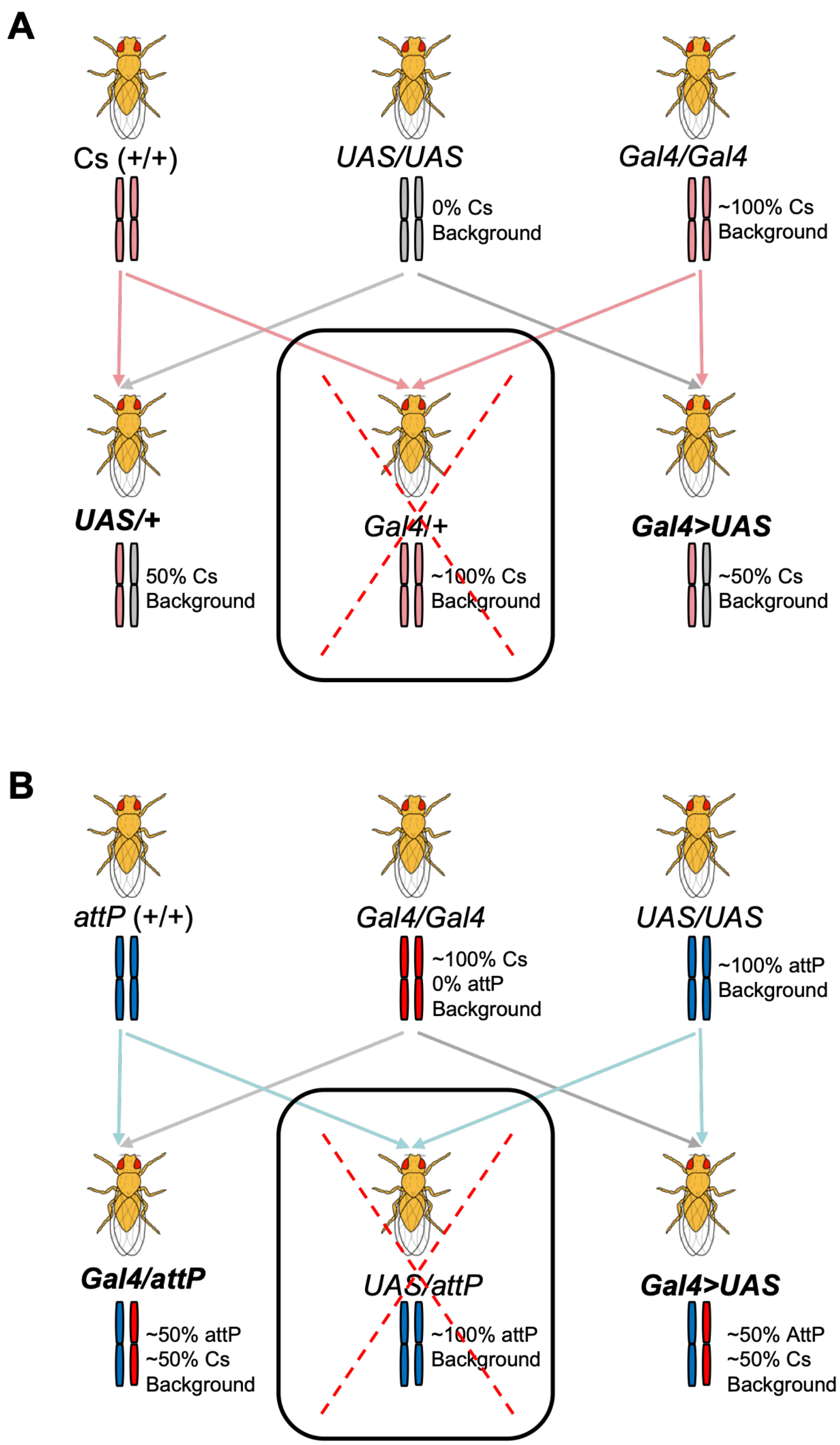

Supplemental Figure 1: Schematic of crossing scheme used to generate genotype controls for all RNAi knockdown experiments. Bolded genotypes are presented in main figures shown. Results for both genetic controls are included in **Supplemental Figures 9-12. (A)** Genetic crossing scheme used for Cs background and exclusion of inappropriate genetic background as a control. **(B)** Genetic crossing scheme used for *attP2* background and exclusion of inappropriate genetic background as a control.

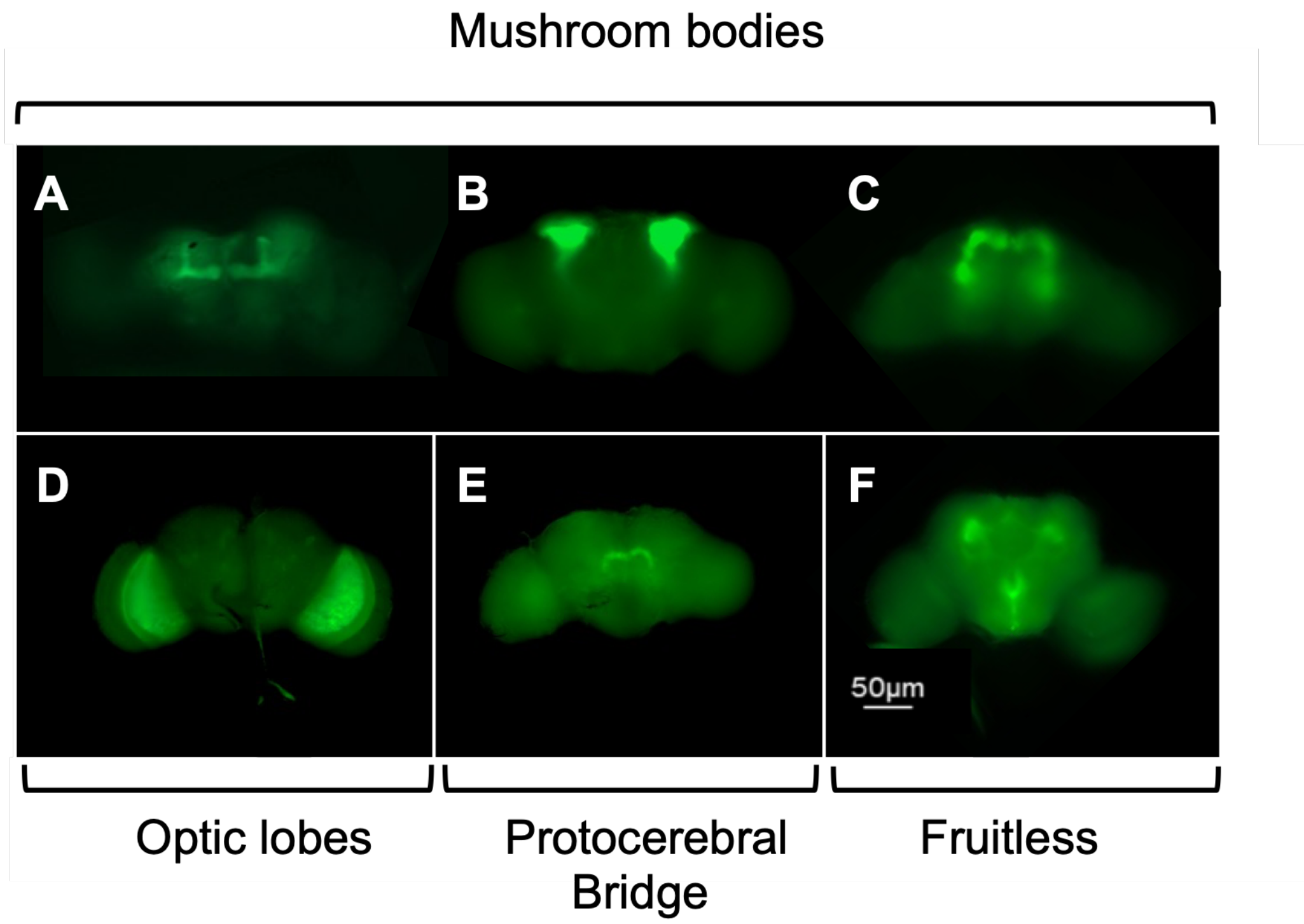

Supplemental Figure 2: Gal-4 Driver confirmations: Representative images of dissected whole adult Drosophila brains (3-4 days old), with the different drivers used in this study, driving membrane-bound GFP: Gal4>UAS-mCD8-GFP, under fluorescent microscope, exposure of 180-200 ms. **(A-C):** different orientations of the mushroom body driver MB>GFP (**A**) anterior/frontal view of the brain with the lobes visible, (**B**) posterior side of the brain where the calyces of the mushroom bodies can be seen. (**C**) overhead view, with both the calyces and peduncles visible. (**D-F**): anterior/frontal views of (**D**) optic lobes driver OL>GFP, (E) protocerebral bridge driver PB>GFP, (F) *fruitless* driver *fruP1*>GFP.

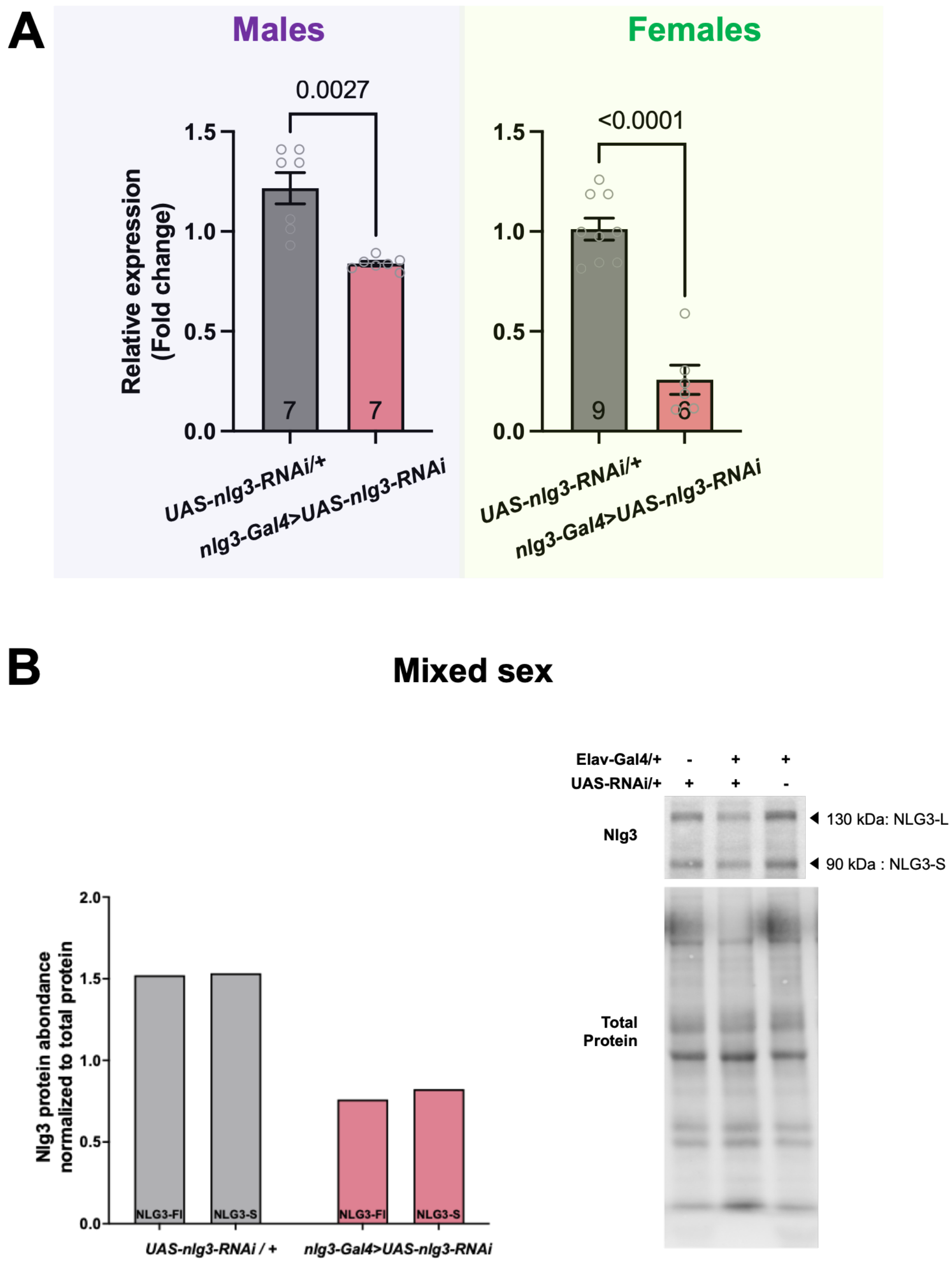

Supplemental Figure 3: Efficacy of UAS-*nlg3* RNAi. (A) Relative expression of *nlg3* mRNA in male (M) and female (F) fly heads (3–5 days old) of either UAS-RNAi *nlg3*/+ or *nlg3*-Gal4>UAS-RNAi *nlg3* measured by qPCR. Data represent mean fold-change (± SEM) from 3 biological replicates with 2-3 technical replicates each, with *Tubulin84* as the reference gene; statistical evidence was assessed using an unpaired Welch t-test. Number of replicates indicated in the bars. **(B) Nlg3 Protein abundance for both isoforms** in mixed sex fly heads (3–5 days old) of either UAS-RNAi *nlg3*/+ or *nlg3*-Gal4>UAS-RNAi *nlg3*. Graph represent abundance relative to total protein levels, from only 1 biological replicate, as we ran out of the anti-body. The graph is the qualitative representation of the picture, which is the original anti-Nlg3 immunoreactivity, performed with one of our last aliquot of the anti-Nlg3 antibody.

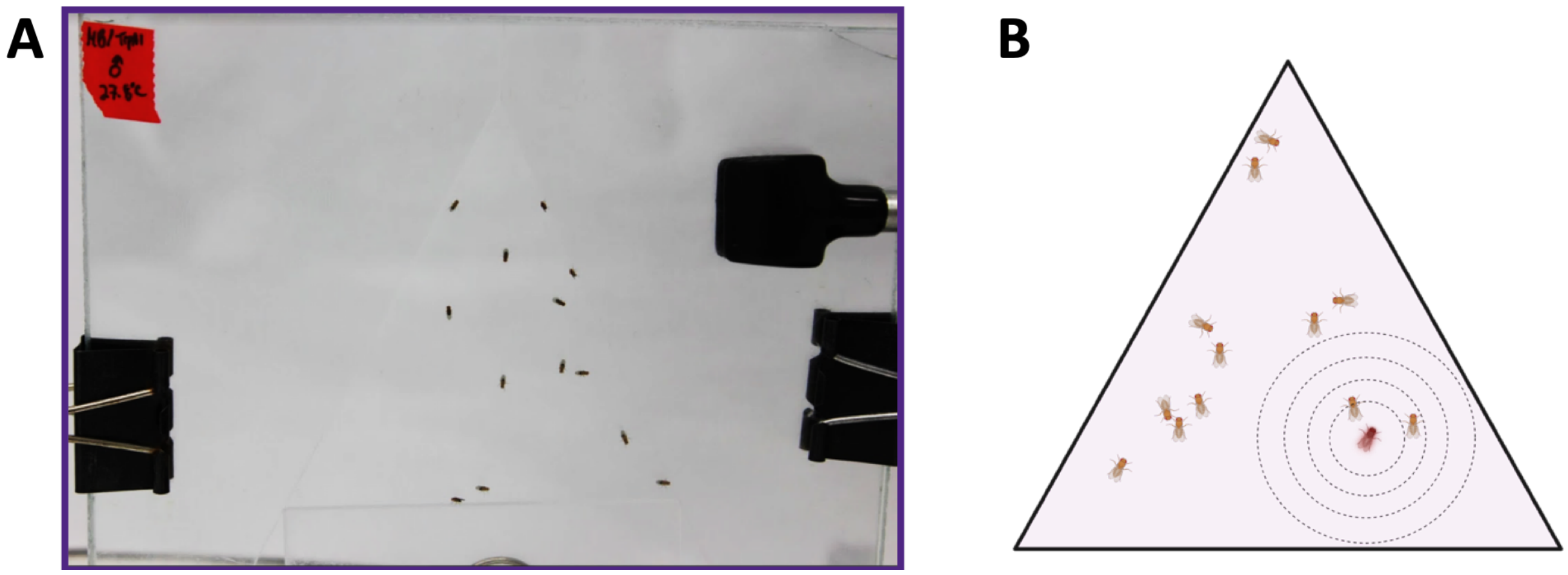

Supplemental Figure 4: Social space chamber 4 body length measurement. (A) Social space chamber constructed using glass panes (17.6 cm by 17.6 cm by 0.3 cm) and acrylic spacers in the shape of right triangles (10.2 cm by 8.9 cm by 8.9 cm, depth of 0.3 cm), accommodating 12-17 flies. (B) Social space is measured by counting the number of flies within a 4-body-length (4BL) radius of a focal fly. In the diagram above, the focal fly (located in the centre of the circle) has a total of two flies within a 4BL radius. This number is measured for each fly in the chamber, and then an average for that chamber is calculated. For more details, see the video associated with the protocol description, in McNeil *et al.* (2015). Created in BioRender. Bechard, A. (2026) https://BioRender.com/v3v0dfk.

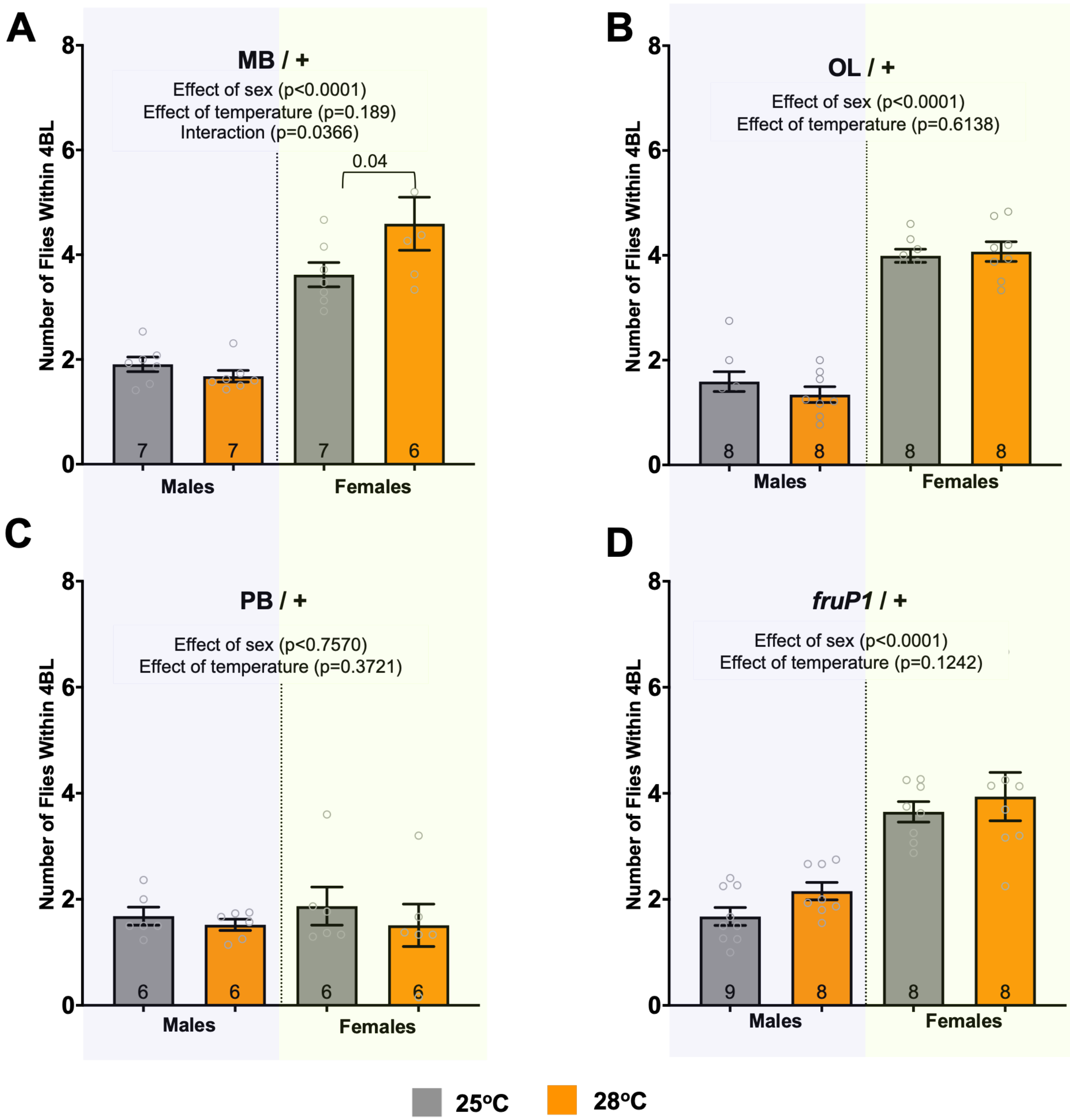

Supplemental Figure 5: Effect of temperature on social space in the heterozygous driver lines used. A-C: Average number of flies within 4 body lengths (4BL) in male and female flies when testing at 25ºC or 28ºC drivers of the MB (A), OL (B), PB (C), or fruP1 (D). The results of all two-way ANOVA performed are shown on the figure. N indicated in the bars for all treatments. Error bars are +/- s.e.m.

Interestingly, in the PB/+ we do not detect an effect of sex, probably because of the influence of that genetic background. Finally, only in the MB/+ do we see a change in social space, with females becoming closer at higher temperature (effect of temperature depending on sex 2-way ANOVA, Interaction between sex and temperature p=0.0366, driven by the females: p=0.04 in a Šidák's post-hoc). However, in our manipulations (**Fig. 3**), we saw that the females driving either *shi*^{ts} or *TrpA1* were further apart, so this effect of temperature on MB/+ is not underlying the changes we report in **Fig. 3**.

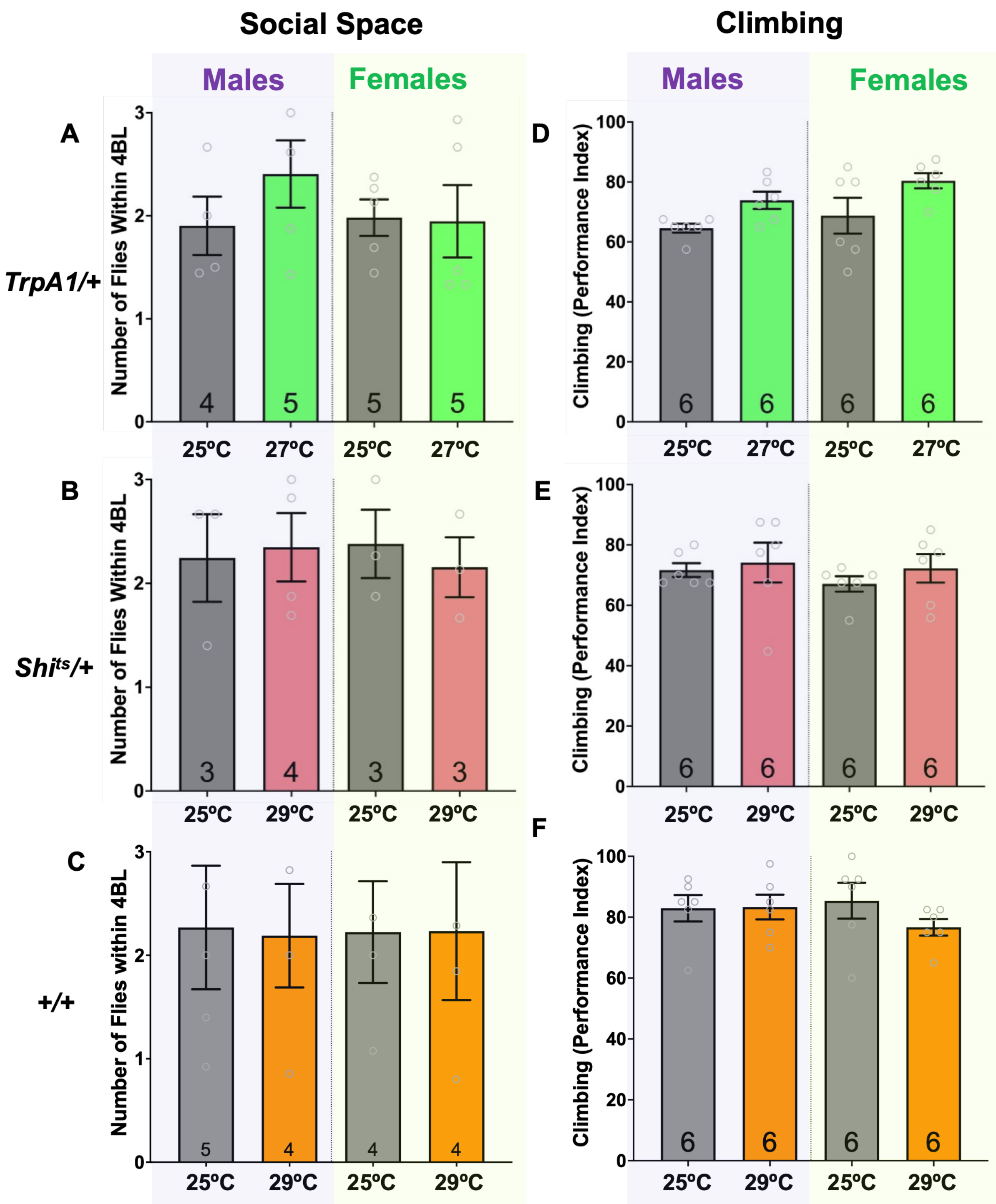

Supplemental Figure 6: Elevated temperature on Cs (+/+), *Trp1A*/+, and *shi*^{ts}/+ does not affect social space or climbing. The genetic controls of the temperature sensitive constructs were tested in social space with the average number of flies within 4 body lengths **A-C:** and climbing with a performance of percentage of flies reaching the top vial **D-F:** in both male and female which reveals the construct alone does not affect social space or climbing. Error bars are +/- s.e.m.

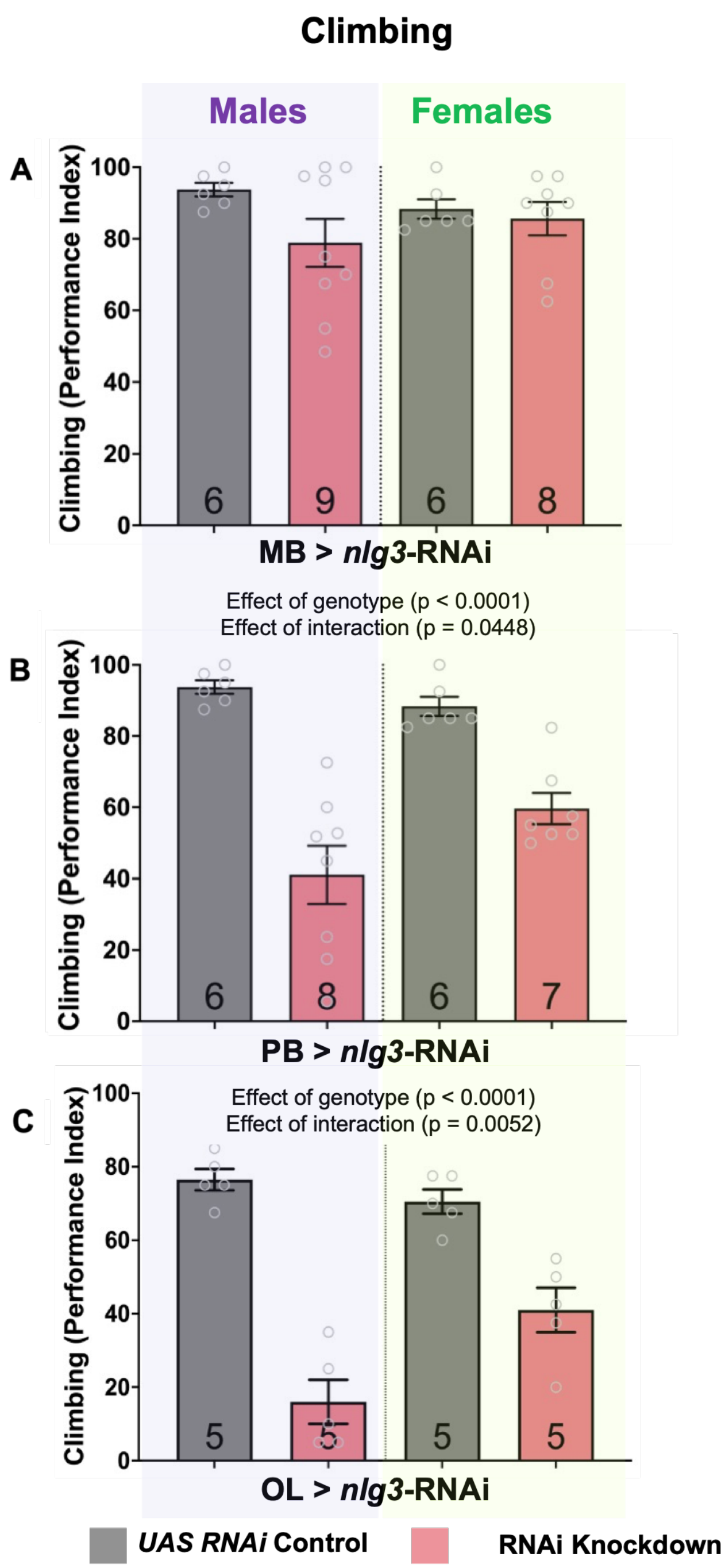

Supplemental Figure 7: Knocking down *nlg3* specifically in the protocerebral bridge and optic lobes decreases fly climbing in males and females but not when knocking down in the mushroom bodies. A-C: Startle-induced climbing represented by the mean percentage of flies reaching the top vial (performance index). Genetic constructs include RNAi knockdown of *nlg3* targeted specifically to the mushroom bodies (A), protocerebral bridge (B), and the optic lobes (C). Grey colour: genetic control *RNAi/+*; pink colour: *driver>RNAi*. N = 5 – 10 for all treatments. Error bars are +/- s.e.m.

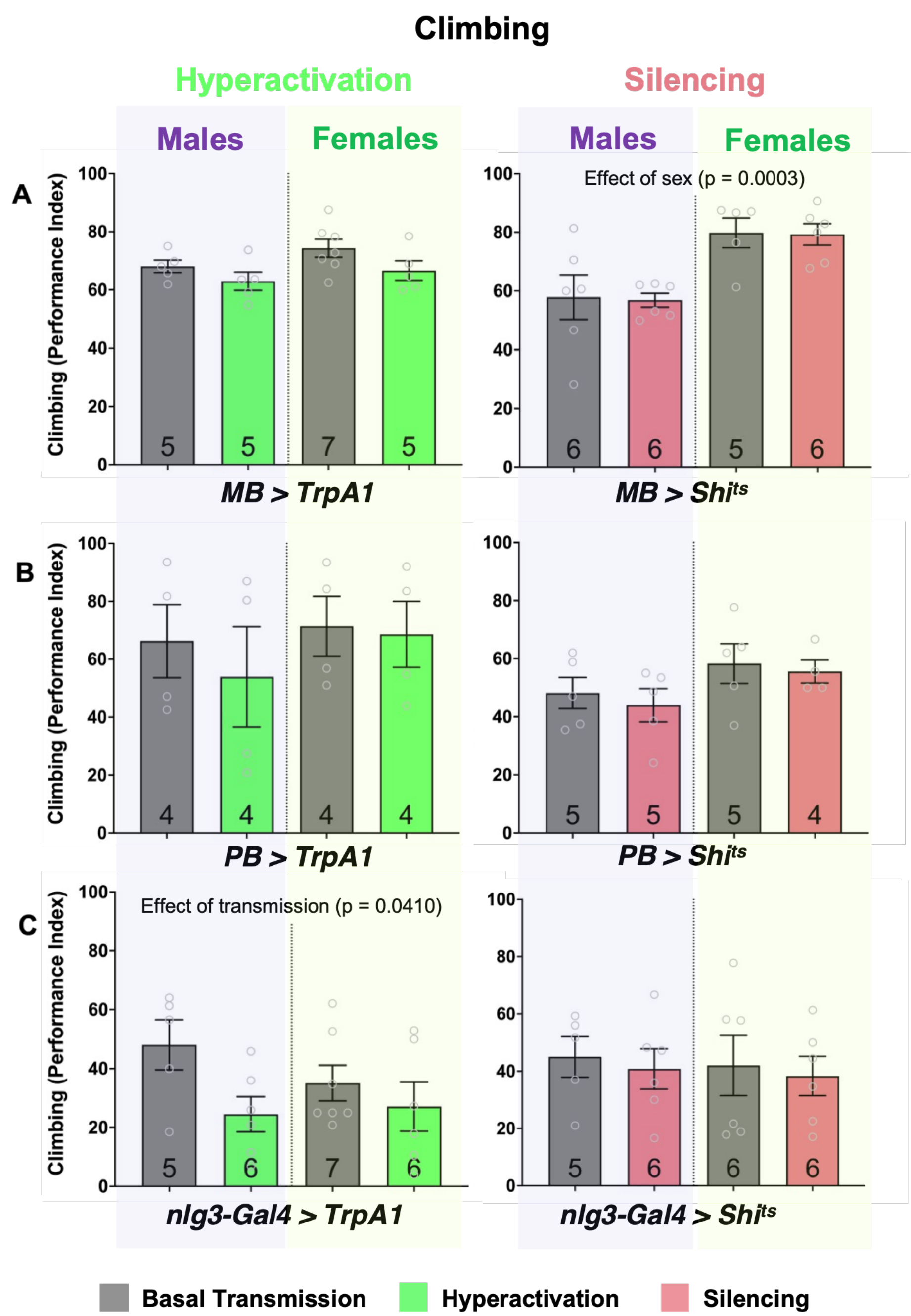

Supplemental Figure 8: Hyperactivation of all the brain structures enriched with Nlg3 decreases fly climbing. A-C: Startle-induced climbing represented by the mean percentage of flies reaching the top vial (performance index). Hyperactivation or silencing neurons specifically to the mushroom bodies (A), protocerebral bridge (B), and all *nlg3*-expressing neurons (C). Grey colour bars: inactivated genetic construct and basal transmission of neurons; green colour bars: activation of genetic construct and hyperactivation of neurons with Gal4 driver; pink colour bars: activation of genetic construct and silencing of neurons with Gal4 driver. The results of all two-way ANOVA performed are shown on the figure. N = 4 – 7 for all treatments. Error bars are +/- s.e.m.

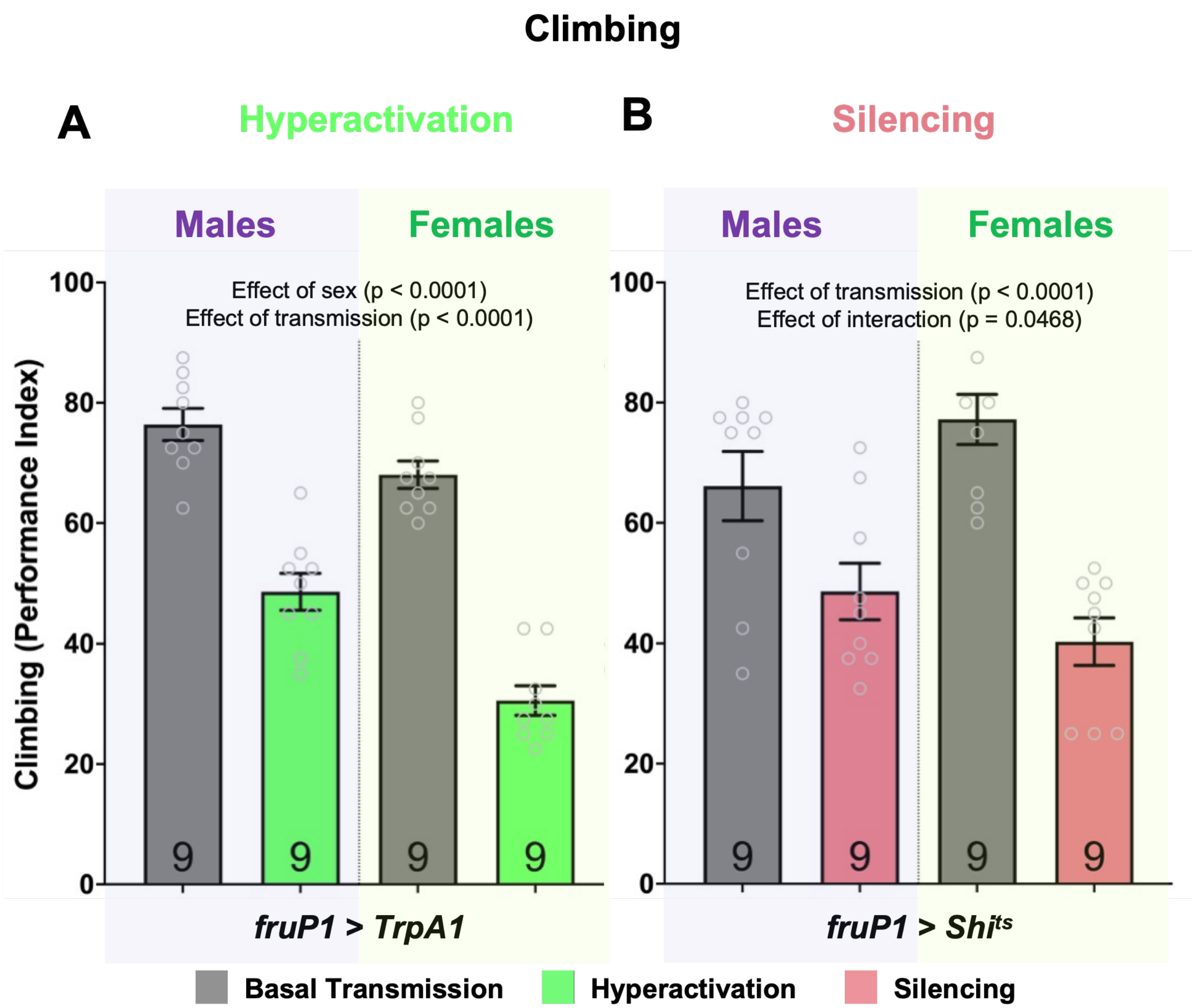

Supplemental Figure 9: Hyperactivation and silencing of *fruP1* neurons decreases climbing in males and females. Startle-induced climbing represented by the mean percentage of flies reaching the top vial (performance index). Hyperactivation or silencing of neurons specifically in the *fruP1* neuron subset. Grey colour bars: inactivated genetic construct and basal transmission of neurons; green colour bars: activation of genetic construct and hyperactivation of neurons with Gal4 driver; pink colour bars: activation of genetic construct and silencing of neurons with Gal4 driver. The results of all two-way ANOVA performed are shown on the figure. N = 9 for all treatments. Error bars are +/- s.e.m.

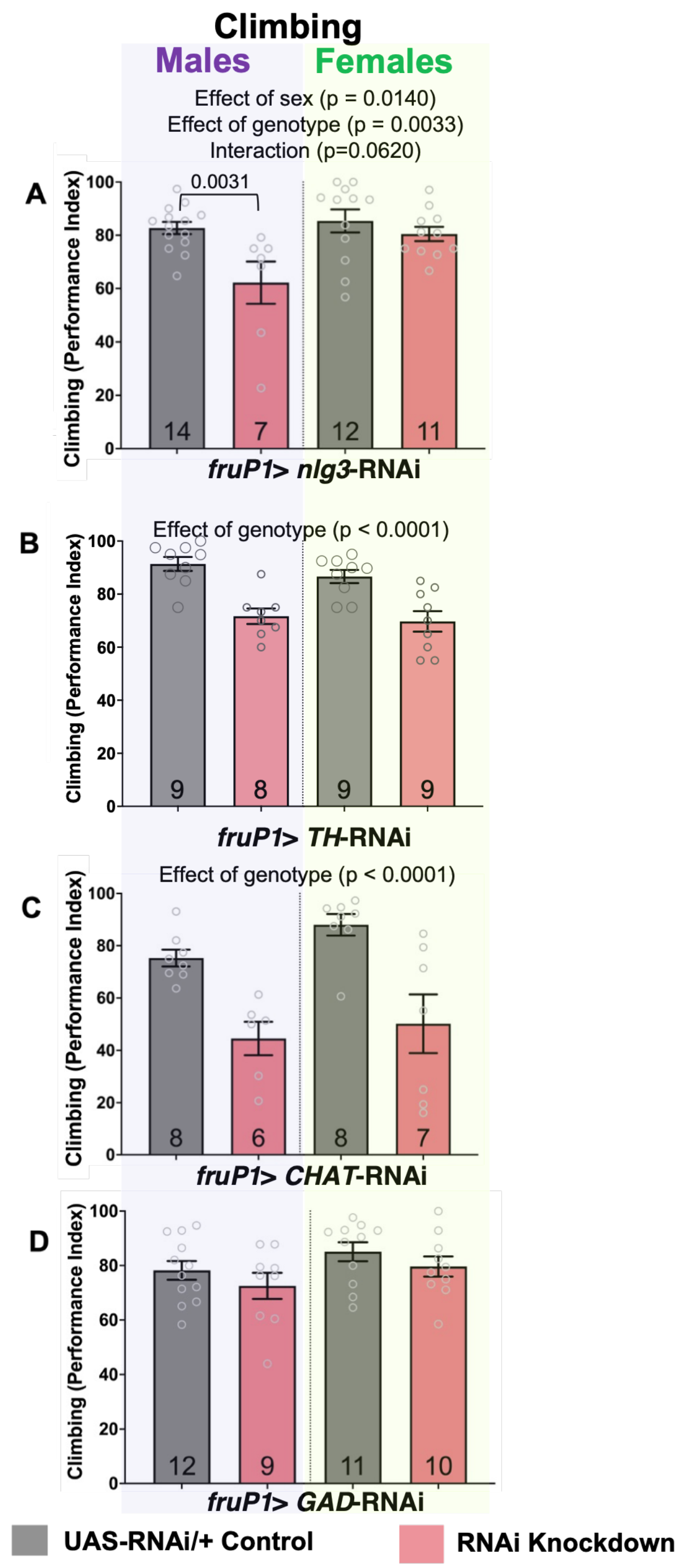

Supplemental Figure 10: Knocking down *nlg3* in *fruP1* neurons decreases male climbing, while a reduction of dopamine, acetylcholine, or GABA in *fruP1* neurons all decrease climbing in male and female flies. A-D: Startle-induced climbing represented by the mean percentage of flies reaching the top vial (performance index). Genetic constructs include RNAi knockdown targeted specifically to the *fruP1* subset of neurons of *nlg3* (A), knocking down dopamine (B), acetylcholine (C), and GABA (D). Grey colour: genetic control *RNAi/+*; pink colour: *driver>RNAi*. The results of all two-way ANOVA performed are shown on the figure. N = 6 – 15 for all treatments. Error bars are +/- s.e.m.

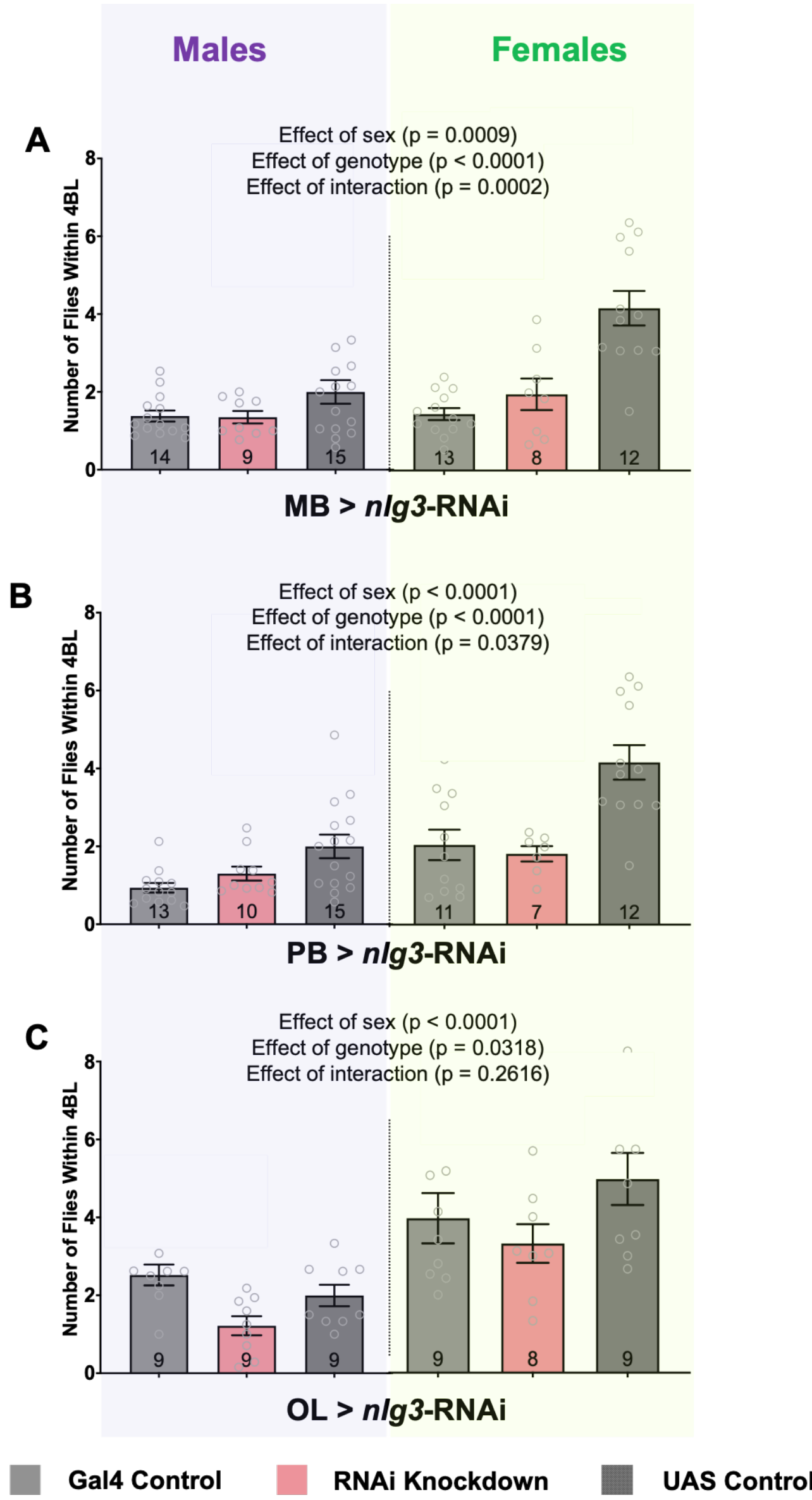

Supplemental Figure 11: Knocking down *nlg3* specifically in the optic lobes increases fly social space in both sexes but no changes are seen when knocking down in the protocerebral bridge or mushroom bodies comparing to both genetic controls with different genetic backgrounds. Average number of flies within 4 body lengths (4BL) in male and female flies with RNAi knockdown of *nlg3* targeted specifically to the mushroom bodies (A), protocerebral bridge (B), and the optic lobes (C). **A.** The number of flies within 4BL does not change in both males and females in flies with *nlg3* knockdown targeted to the MB. Comparing the knockdown line to both control lines shows an intermediate phenotype. **B.** The average number of flies within 4BL does not change in both males and females in flies with *nlg3* knockdown targeted to the PB. Comparing the knockdown line to both control lines shows an intermediate phenotype. **C.** Knocking down *nlg3* in the OL decreases the number of flies within 4BL in both males and females compared to genetic control flies (two-way ANOVA – Effect of genotype: $p = 0.0318$). Grey colour: genetic control *RNAi/+* and *driver/+*; pink colour: *driver>RNAi*. N = 7 – 14 for all treatments. Error bars are +/- s.e.m.

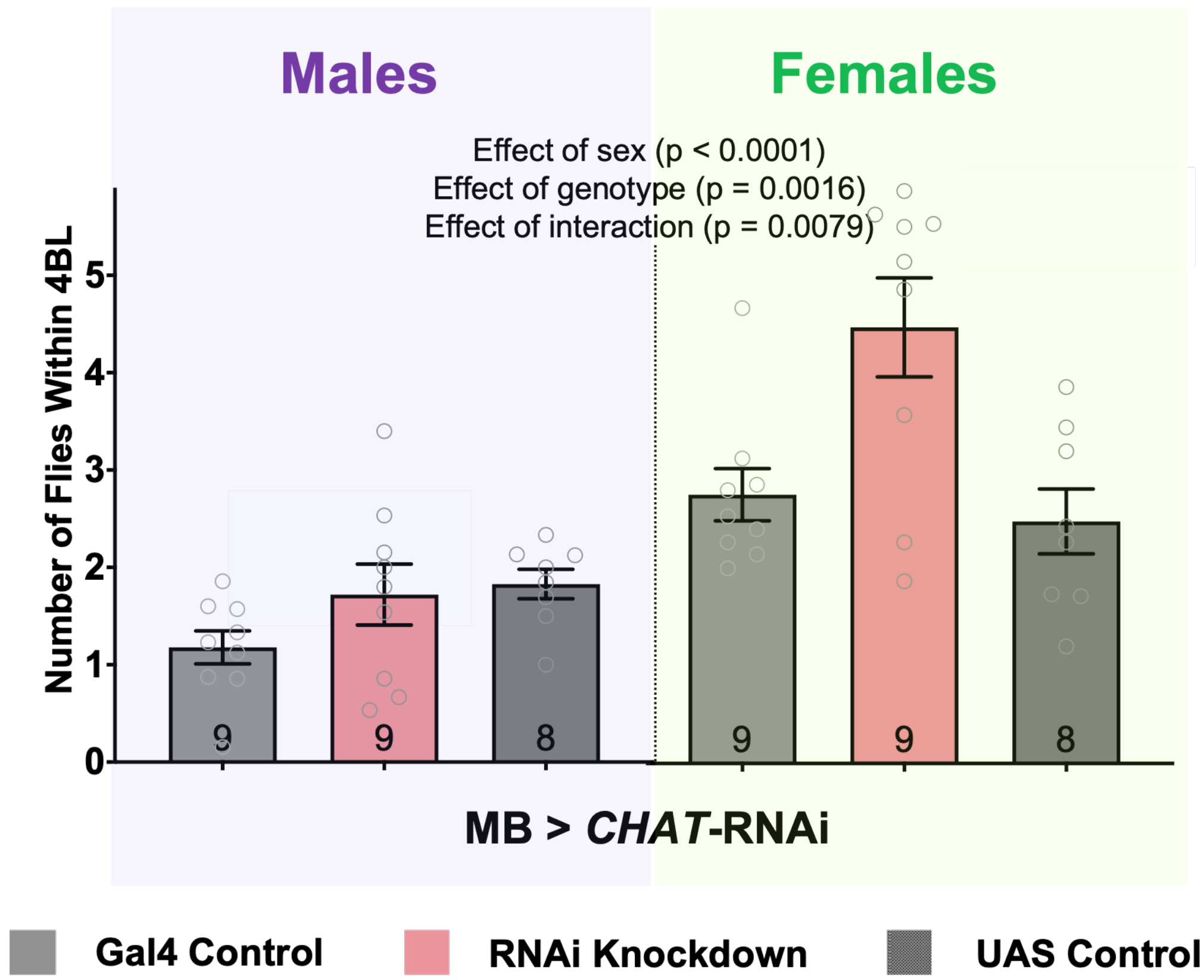

Supplemental Figure 12. Knocking down the acetylcholine rate-limiting biosynthesis enzyme C*hAT* in the MB decreases female social space when comparing to both genetic controls with different genetic backgrounds. Average number of flies within 4 body lengths (4BL) in male and female flies with RNAi knockdown of *ChAT* for acetylcholine synthesis specifically in the mushroom bodies. The interaction between sex and *ChAT* knockdown shows that female flies have an increased number of flies within 4BL when compared to both the genetic control flies (two-way ANOVA – Effect of interaction: $p = 0.0079$). Comparing the female *ChAT* knockdown line to both control lines shows an intermediate phenotype. Grey colour bars: genetic control *driver/+* and *driver/+*; pink colour bars: *driver>RNAi*. N = 8 – 9 for all treatments. Error bars are +/- s.e.m.

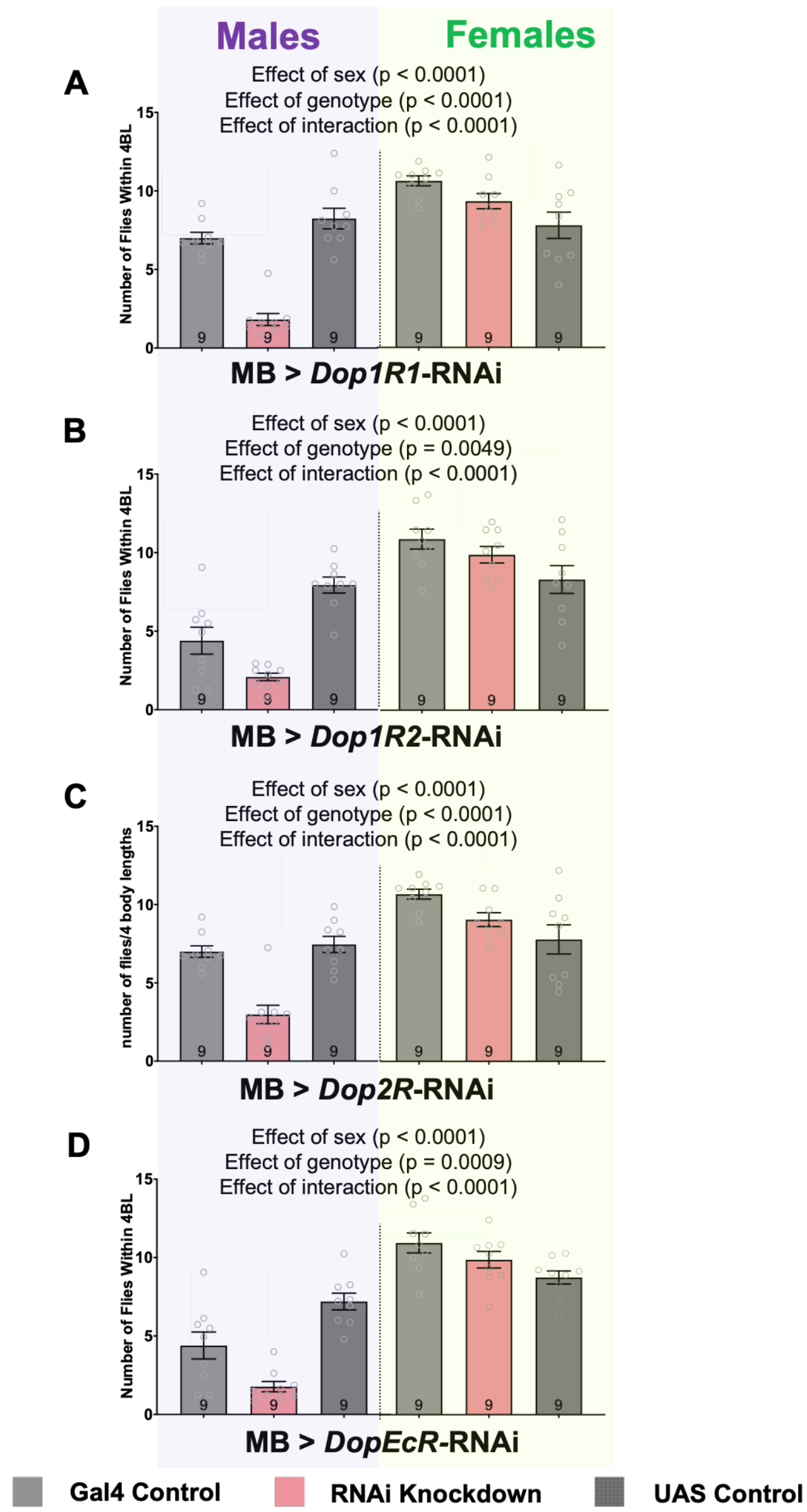

Supplemental Figure 13: RNAi manipulation of all dopamine receptors in the MB increases male social space when comparing with both genetic controls with different genetic backgrounds. The main conclusions remain unchanged when comparing with both genetic controls from the V10 background even with different genetic backgrounds. Average number of flies within 4 body lengths (4BL) in male and female flies knocking down dopamine receptors show that *Dop receptors* knockdown in the MB of males has a decreased number of flies within 4BL compared to genetic controls. **A.** *Dop1R1* (Two-way ANOVA – Effect of genotype: $F_{1,32} = 19.61$, $p < 0.0001$). **B.** *Dop1R2* (Two-way ANOVA – Effect of genotype: $F_{1,32} = 41.10$, $p < 0.0001$). **C.** *Dop2R* (Two-way ANOVA – Effect of genotype: $F_{1,32} = 41.10$, $p < 0.0001$). **D.** *DopEcR* (Two-way ANOVA – Effect of genotype: $F_{1,32} = 8.905$, $p = 0.0054$). Grey colour bars: genetic control *driver/+* and *Gal4/+*; pink colour bars: *driver>RNAi*. The results of all two-way ANOVA performed are shown on the figure. N = 9 for all treatments. Error bars are +/- s.e.m.

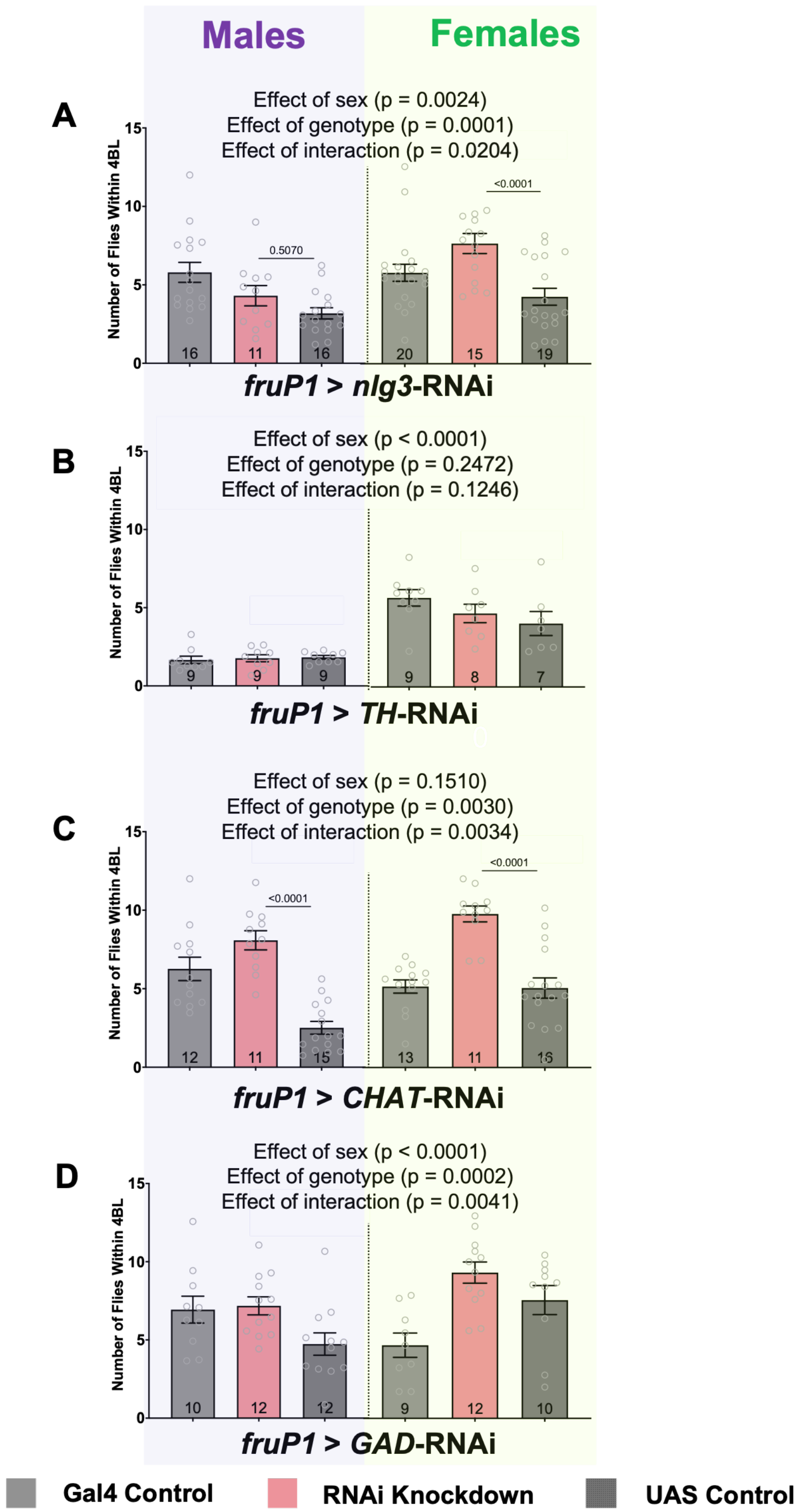

Supplemental Figure 14: Knocking down *nlg3* in *fruP1* neurons decreases female social space, while a reduction of acetylcholine in *fruP1* neurons decreases social space in both sexes when comparing with both genetic controls with different genetic backgrounds. Average number of flies within 4 body lengths (4BL) in male and female flies with RNAi knockdown of *nlg3* (A), *TH* for dopamine synthesis (B), *ChAT* for acetylcholine synthesis (C) or *GAD1* for GABA synthesis (D) specifically in *fruP1* neurons. **A.** The interaction between sex and *nlg3* knockdown shows that female flies have a decreased number of flies within 4BL when compared to both the genetic control flies (two-way ANOVA – Effect of interaction: $p = 0.0204$). Comparing the male *nlg3* knockdown to both control lines shows an intermediate phenotype. **B.** *TH* knockdown in *fruP1* neurons does not change social space in males and females. **C.** *ChAT* knockdown in *fruP1* neurons increases the number of flies within 4BL in both males and females compared to the genetic control flies (two-way ANOVA – Effect of genotype: $p = 0.0030$). **D.** The interaction between sex and *GAD1* knockdown shows that female flies have an increased number of flies within 4BL when compared to both the genetic control flies (two-way ANOVA – Effect of interaction: $p = 0.0041$). Male *GAD1* knockdown in *fruP1* neurons shows an intermediate phenotype when compared to both genetic controls. Grey colour bars: genetic control *RNAi/+* and *driver/+*; pink colour bars: *driver>RNAi*. The results of all two-way ANOVA performed are shown on the figure. N = 9 - 13 for all treatments. Error bars are +/- s.e.m.
